# Regulation of N-WASP and the Arp2/3 Complex by Abp1 Controls Neuronal Morphology

**DOI:** 10.1371/journal.pone.0000400

**Published:** 2007-05-02

**Authors:** Roser Pinyol, Akvile Haeckel, Anett Ritter, Britta Qualmann, Michael Manfred Kessels

**Affiliations:** 1 Research Group Membrane Trafficking and Cytoskeleton, Department of Neurochemistry and Molecular Biology, Leibniz Institute for Neurobiology, Magdeburg, Germany; 2 Research Group Cell Biology, Leibniz Institute for Neurobiology, Magdeburg, Germany; Max Planck Institute of Molecular Cell Biology and Genetics, Germany

## Abstract

Polymerization and organization of actin filaments into complex superstructures is indispensable for structure and function of neuronal networks. We here report that knock down of the F-actin-binding protein Abp1, which is important for endocytosis and synaptic organization, results in changes in axon development virtually identical to Arp2/3 complex inhibition, i.e., a selective increase of axon length. Our in vitro and in vivo experiments demonstrate that Abp1 interacts directly with N-WASP, an activator of the Arp2/3 complex, *and* releases the autoinhibition of N-WASP in cooperation with Cdc42 and thereby promotes N-WASP-triggered Arp2/3 complex-mediated actin polymerization. In line with our mechanistical studies and the colocalization of Abp1, N-WASP and Arp2/3 at sites of actin polymerization in neurons, we reveal an essential role of Abp1 and its cooperativity with Cdc42 in N-WASP-induced rearrangements of the neuronal cytoskeleton. We furthermore show that introduction of N-WASP mutants lacking the ability to bind Abp1 or Cdc42, Arp2/3 complex inhibition, Abp1 knock down, N-WASP knock down and Arp3 knock down, all cause identical neuromorphological phenotypes. Our data thus strongly suggest that these proteins and their complex formation are important for cytoskeletal processes underlying neuronal network formation.

## Introduction

The organization and dynamics of the cortical actin cytoskeleton play important roles in cell migration, establishment and changes of cell morphology and adhesion but also in cellular uptake processes, such as phagocytosis, macropinocytosis and receptor-mediated endocytosis [1]–[5] – processes, which are indispensable for individual cells, the formations of larger cellular networks and organogenesis.

The importance of actin filament polymerization and dynamics in neuronal cells has been primarily investigated during neuronal development. The formation and migration of growth cones and neurites but also the establishment of neuronal polarity critically relies on actin dynamics [6]. Furthermore, cytoskeletal elements play an important role in the structural and functional organization of the pre- and the postsynaptic compartment [7]–[8].

A powerful nucleator of actin filaments is the actin-related protein 2 and 3 (Arp2/3) complex. Purified Arp2/3 complex alone poorly nucleates actin filaments and thus requires nucleation-promoting factors for efficient activity [9]–[10]. Since the Arp2/3 complex is involved in diverse actin cytoskeletal functions, specificity for individual processes and morphological features can only be brought about by distinct regulatory pathways and by further accessory components of this core actin nucleation machinery. One of the most prominent Arp2/3 complex activators is the neural Wiskott-Aldrich syndrome protein (N-WASP; gi|2274845) [11], which exhibits the highest expression levels in the brain [12]. N-WASP is kept in an autoinhibited state through an intramolecular association [13] preventing the interaction of its C-terminus with the Arp2/3 complex [14]. Several factors are suggested to control the activity of WASP proteins by promoting its conversion from an inactive to an active form [10], [15]–[16]. Although the in vivo evidence for Arp2/3-complex-dependent functions of a majority of these factors is sparse, their diversity may offer opportunities for cooperative regulations and may, in time and space, define the linkage of Arp2/3 complex-mediated actin nucleation machinery to different cellular processes relying on actin polymerization.

Our previous studies suggested a role for the mammalian F-actin-binding protein 1 (Abp1; SH3P7; gi|1407655; gi|51315733) in Arp2/3 complex-mediated actin dynamics. Abp1 does not associate with relatively static actin structures but strongly accumulates at the dynamic leading edge of moving and spreading cells where it colocalizes extensively with the Arp2/3 complex. The occurrence of Abp1 at sites of high actin dynamics is dependent on de novo actin polymerization and controlled by different signaling cascades, such as activation of Rho-type GTPases. In vitro-reconstitution assays with actin and recombinant Abp1 had, however, not revealed any direct effects on actin dynamics [17]. We here describe that knock down of Abp1, which has furthermore been characterized as a component important for receptor-mediated endocytosis [18]–[20] and synaptic organization [21]–[22], affects neuronal morphology in a manner that is virtually identical to Arp2/3 complex inhibition [23]. Aimed to unravel the underlying molecular mechanisms, our experiments show that Abp1 directly interacts with N-WASP and releases its autoinhibition. Abp1 thereby modulates N-WASP-mediated Arp2/3 complex-triggered actin filament polymerization and plays an important role in neuronal morphology control.

## Results

### Reduced Abp1 expression levels selectively result in increased axon length in early stages of neuronal development

**Figure 1:**
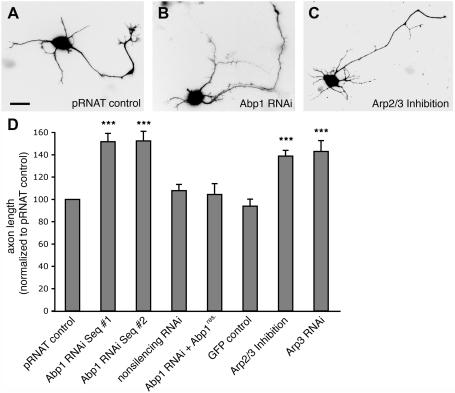
Abp1 knock down selectively results in increased axon length. (A–D) Primary hippocampal neurons transfected with pRNAT-GFP (control; A), with a pRNAT-driven Abp1 RNAi construct (sequence #1; B), and with GFP-N-WASP CA (Arp2/3 complex inhibition; C), respectively, at day 2 in culture. The cells were fixed and processed for immunofluorescence microscopy at day 3. Quantitative evaluations (D) show that neurons (as evidenced by MAP2 staining) expressing the pRNAT-driven Abp1 RNAi sequences #1 and #2 both show highly significantly (p<0.001, ***) elongated axons when compared to controls (transfection with pRNAT, pEGFP and nonsilencing RNAi) and to cells in rescue experiments, in which Abp1 RNAi and an RNAi-insensitive Abp1 construct were cotransfected. Effects almost identical to those caused by Abp1 RNAi were observed when Arp2/3 complex functions were impaired by overexpression of GFP-N-WASP CA (C, D) and Arp3 RNAi (D), respectively. Morphological data were normalized to pRNAT-control and are represented as mean (in percent)±SEM. Bar = 10 µm.

The mammalian cytoskeletal component Abp1 directly associates with F-actin [17]. In neurons, cells, whose structure, polarity and functionality critically relies on actin filament organization and dynamics, Abp1 is present at considerable high levels already early in development and in both the axonal and the dendritic compartment [17], [21]–[22]. A potential critical role of Abp1 in morphology control in neurons was therefore addressed by applying RNA interference. Two different RNA interference (RNAi) sequences employed very similarly yielded an almost 60% decrease in the fluorescence intensity of Abp1 immunodetection (Figure S1) and were thus used for further experiments. In comparison to control cells (Figure 1A), developing neurons with reduced Abp1 expression levels (Figure 1B) showed a prominent increase in axon length. Quantitative examinations (Figure 1D) revealed that axons lengths from Abp1-deficient neurons were in average 151.7±7.4% (Abp1 RNAi sequence #1) and 152.5±8.5% (Abp1 RNAi sequence #2) of control values (pRNAT-transfected neurons). The phenotype was specifically caused by Abp1 deficiency because cotransfecting the cells with an Abp1 construct carrying several silent mutations, which render the mRNA resistant to RNAi, restored the wild-type situation (Figure 1D). In contrast, the length of dendrites and the number of neurites per cell were almost unaffected by Abp1 knock down (data not shown). This very specific effect on neuronal morphology caused by Abp1 was virtually identical to Arp2/3 complex inhibition through overexpression of green fluorescent protein (GFP)-N-WASP CA (Figure 1C, [23]). In our hands, axon length from neurons with Arp2/3 complex inhibition increased to 138.8±5.2% of control (Figure 1D). These data were corroborated by actin-related protein 3 (Arp3; gi 11037751) RNAi. For this purpose we used an RNAi targeting sequence established by Steffen et al. [24]. Arp3 RNAi led to neurons exhibiting significantly elongated axons as well. Quantitative examinations demonstrated that Arp3 knock down led to an average axon length of 143.0±9.7% of control (Figure 1D). These data were thus indistinguishable from those for Abp1 knock down and for Arp2/3 complex inhibition by N-WASP CA overexpression (Figure 1D).

### Abp1 stimulates actin polymerization in an Src homology 3 (SH3) domain-dependent manner *in vitro*

**Figure 2:**
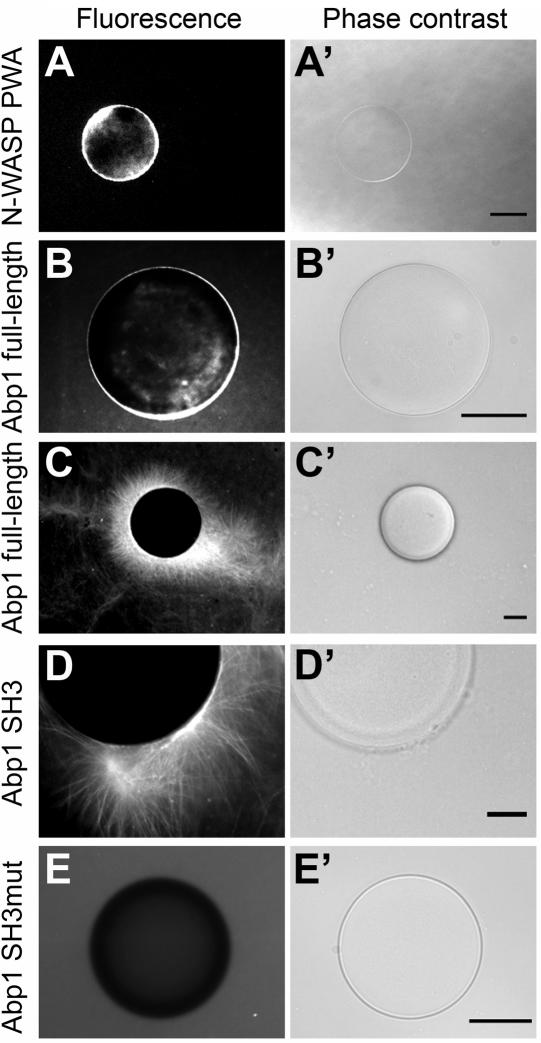
Full-length Abp1 and its SH3 domain alone both stimulate actin polymerization in vitro. GST-fusion proteins attached to glutathione sepharose 4B beads were incubated with high speed supernatants of brain extracts supplemented with an energy regenerating mix and Alexa Fluor® 568 G-actin. Actin polymerization is detected on the surface of beads coated with N-WASP PWA (A), full-length Abp1 (B, C) and the Abp1 SH3 domain (D). No polymerization was observed when the beads were coated with a mutated version of the Abp1 SH3 domain (E). Left panels (A–E), actin fluorescence of Alexa Fluor® 568 G-actin; right panels (A′–E′), phase contrast. Bars (A and D) = 10 µm; bars (B, C and E) = 25 µm.

The striking similarity of the effects of Abp1 knock down and Arp2/3 complex inhibition on neuronal morphology strongly suggested that Abp1 functions are tightly connected to Arp2/3 complex functions. We therefore tested whether Abp1 is capable to promote actin polymerization by incubating beads coated with recombinant proteins with rat brain extracts supplemented with an energy regenerating mix and fluorescently labeled G-actin. The assay was set up using the extended C-terminal part of N-WASP (N-WASP PWA), which interacts with and stimulates the Arp2/3 complex, as positive control. Glutathione S-transferase (GST)-N-WASP PWA led to fluorescent halos on the beads corresponding to newly polymerized actin (Figure 2A), while GST used as negative control did not (data not shown). Very similar to GST-N-WASP-PWA (Figure 2A), GST-fusion proteins of full-length Abp1 immobilized on the bead surface induced actin structures (Figure 2B and 2C). Besides halos (Figure 2B), occasionally, even extended F-actin networks originating from Abp1-coated beads were observed (Figure 2C).

Since full-length Abp1 contains two N-terminal F-actin binding domains [17], we could not exclude that the observed fluorescent structures were actin filaments that were merely recruited to the beads. Additional bead assays were therefore performed with the C-terminal Abp1 SH3 domain. This domain is incapable to directly associate with actin [17] but similar to our experiments with full-length Abp1, fluorescent actin halos and occasionally even extended actin structures were also formed on beads coated with the Abp1 SH3 domain (Figure 2D). The effect was specific, because neither GST (data not shown) nor beads coated with a mutated SH3 domain (P422L and G425R) incapable of undergoing classical SH3 domain interactions with PXXP-motifs [18] were able to induce F-actin structures (Figure 2E). We thus concluded that the Abp1 SH3 domain is capable to and sufficient for triggering actin nucleation.

### Abp1 colocalizes with the Arp2/3 complex and its activator N-WASP at sites of actin polymerization in hippocampal neurons

**Figure 3:**
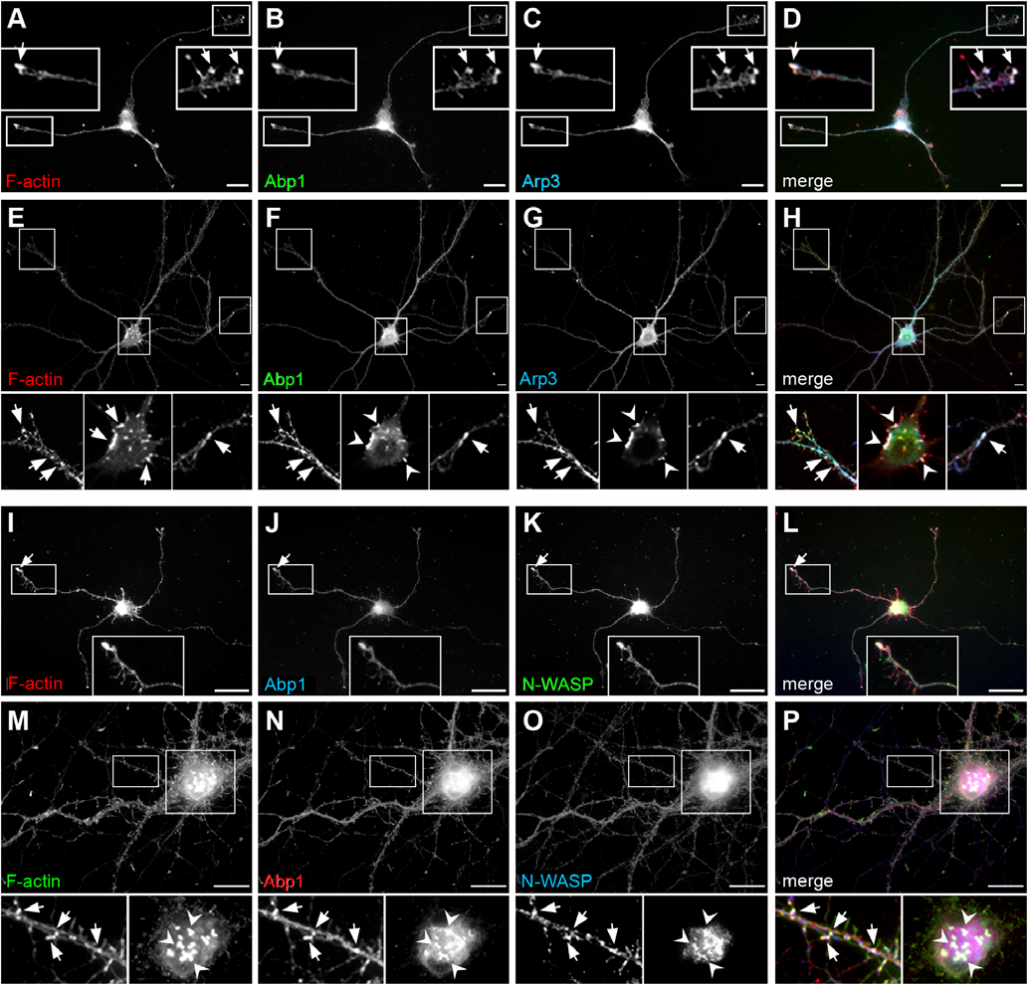
Abp1, Arp3 and N-WASP colocalize in young and mature neurons at sites of actin polymerization. 4 d.i.v. (A–D, I–L) and 21 d.i.v. (E–H, M–P) primary hippocampal neurons were immunostained with anti-Abp1 antibodies (B, F, J, N), anti-Arp3 antibodies (C, G), anti-N-WASP antibodies (K, O) and phalloidin, to visualize F-actin (A, E, I, M). Labelling of images reflects the color of the fluorescence signal in the merged images (D, H, L, P; colocalization appears white). Colocalization at growth cones from young neurons (D, L) and at sites within the periphery of the neuronal network from mature neurons that may represent synaptic contacts (H, P) are magnified and marked with arrows. In mature neurons, Abp1 (F, N), N-WASP (O), as well as the Arp2/3 complex (G) were additionally observed to colocalize at actin-rich structures (E, M) of the cell body and dendritic shafts, as marked by arrowheads in the magnified insets. Bars = 25 µm.

Supporting a role for Abp1 in Arp2/3 complex-mediated control of neuronal morphology, colocalization studies show a high degree of overlap for the immunoreactivities of Arp3 with that of Abp1 in hippocampal neuronal cultures at different stages of development. Both proteins accumulate at sites enriched for polymerized F-actin (Figure 3A–H). In young neurons (4 days in vitro (d.i.v.)), Abp1 displayed a relatively uniform localization in the cell soma and in neurites with prominent accumulations at F-actin-rich growth cones and in the perinuclear area (Figure 3B). The observed perinuclear localization of Abp1 is consistent with its role in Golgi trafficking [25]. The high abundance of Abp1 and Arp3 in growth cones (Figure 3A–D) is consistent with the observed role of these components in regulating axon extension.

In mature neurons (21 d.i.v.), Abp1 showed a spatially well-defined staining at sites within the periphery of the neuronal network (arrows in magnifications; Figure 3F) that are likely to represent synaptic contacts [22]. The staining patterns of Arp3 and F-actin were similar to that of Abp1 (Figure 3E–H). Additionally, Arp3 strongly accumulated in characteristic Abp1-positive and F-actin-rich structures in the cell bodies and in dendritic shafts (Figure 3E–H). These colocalizations are in line with the hypothesis that Abp1 plays a role in actin dynamics mediated by the Arp2/3 complex.

### Abp1 interfaces with the Arp2/3 complex indirectly by interacting with N-WASP

**Figure 4:**
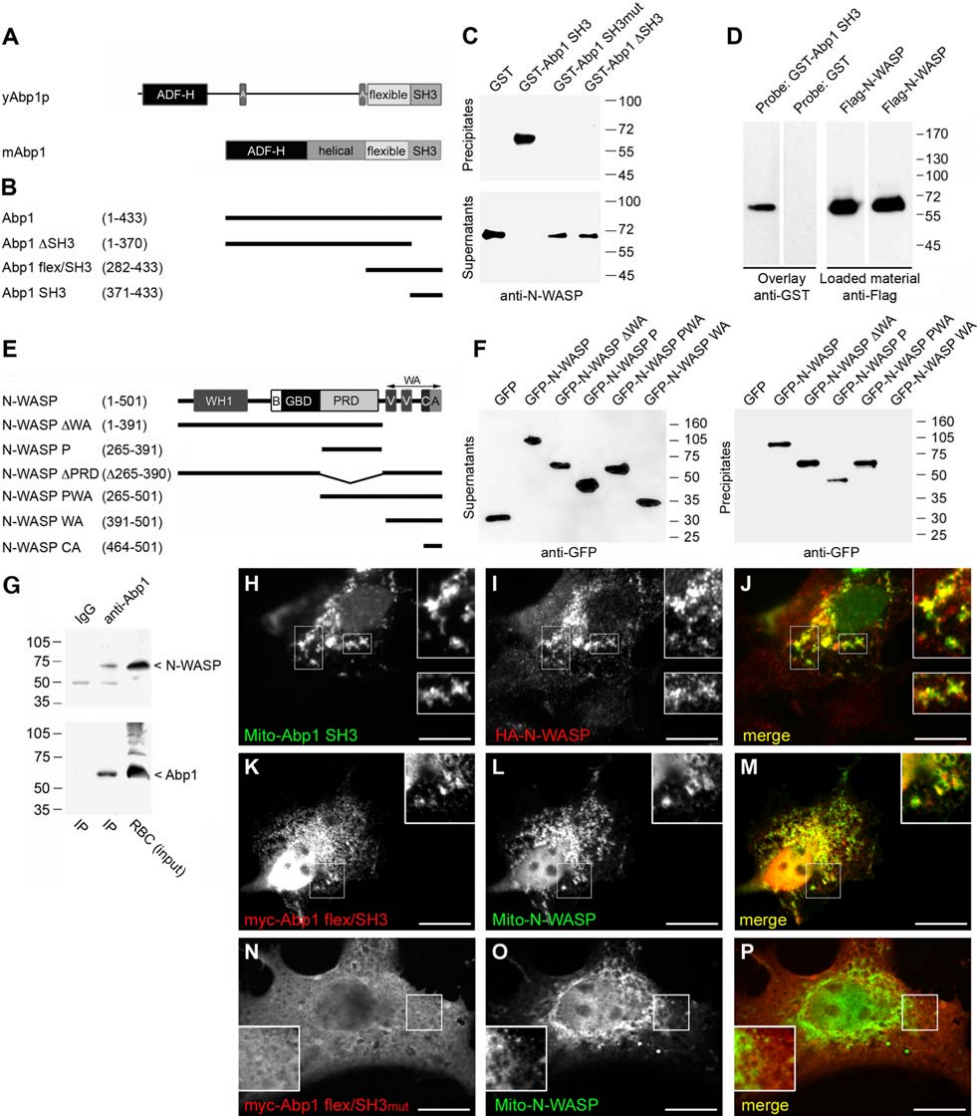
Abp1 interacts specifically and directly with N-WASP and such Abp1/N-WASP complexes exist in vivo. (A) Comparison of the domain structure of yeast Abp1p (yAbp1p) and mouse Abp1 (mAbp1/SH3P7) proteins. (B) Schematic representation of the mouse Abp1 fragments used throughout this study. (C) Immobilized GST-Abp1 SH3 domain is required and sufficient to affinity purify endogenous N-WASP from rat brain cytosol. (D) Blot overlay analyses. 1.5 µg affinity-purified Flag-tagged full-length N-WASP blotted to nitrocellulose membranes was directly bound by GST-Abp1 SH3 but not by GST probes (left panel). N-WASP was additionally visualized by anti-Flag immunostaining (right panel). (E) Schematic representation of the N-WASP fragments used throughout this study. (F) Affinity purifications of overexpressed GFP-tagged N-WASP and fragments thereof with immobilized GST-Abp1 SH3 domain reveal that Abp1 associates with N-WASP fusion proteins containing the PRD. (G) Endogenous N-WASP was specifically coimmunoprecipitated with endogenous Abp1. An equal amount of brain extract was subjected to immunoprecipitation with unrelated rabbit IgGs as a control. (H–P) HA-N-WASP (I) was corecruited by the Mito-GFP-Abp1 SH3 domain (H) to mitochondria. (K-P) Mitochondrially targeted N-WASP recruits Abp1 in an SH3 domain-dependent manner. Upon coexpression of Mito-GFP-N-WASP (L, O), wild type Abp1 C-terminus (flex/SH3) adopted a mitochondrial localization (K) whereas its mutated version flex/SH3mut was distributed diffusely (N) and did not colocalize with mitochondria enriched for N-WASP (O). Labelling of images reflects the color of the fluorescence signal in the merged images (J, M and P; colocalization appears yellow). Bars = 15 µm.

In contrast to yeast Abp1p (gi|113000), mammalian Abp1 does not exhibit any motif typical for direct Arp2/3 complex binding and activation (Figure 4A). Consistent with this lack of conservation of the motifs that mediate a direct interaction of yeast Abp1p with the Arp2/3 complex [26], we did not observe any direct binding of Abp1 to the Arp2/3 complex in a variety of biochemical experiments, including affinity purifications with different immobilized Arp2/3 complex components and yeast-two hybrid analyses (data not shown).

In order to unravel the molecular mechanism underlying the observed effects of Abp1 knock down on axon development, we therefore tested the hypothesis of an indirect association with the Arp2/3 complex and of a potential activity modulation of the Arp2/3 complex by an Abp1 interaction with an Arp2/3 complex activator. Immunocytochemical analyses revealed that Abp1 does not only colocalize with the Arp2/3 complex but also with its catalytic activator N-WASP at growth cones from young neurons (Figure 3I–L) and at synaptic contact sites within the periphery of the neuronal network from mature neurons (Figure 3M–P). This suggested that Abp1 might interface with the Arp2/3 complex via N-WASP. Since our studies revealed that the Abp1 SH3 domain is mediating interactions with the cellular actin polymerization machinery (Figure 2), we used GST-fusion proteins of the SH3 domain of Abp1 (Figure 4B) in coprecipitation experiments with rat brain extracts and could indeed identify the Arp2/3 complex activator N-WASP as Abp1-binding partner (Figure 4C).

Additional experiments with different parts and mutants of Abp1 revealed that in line with the in vitro reconstitution data of actin polymerization (Figure 2) the Abp1 SH3 domain is required and sufficient for N-WASP binding. Whereas the immobilized SH3 domain of Abp1 quantitatively affinity purified endogenous N-WASP from brain extracts, both Abp1 ΔSH3 and a mutated version of the SH3 domain (P422L and G425R; [18]) showed no affinity for N-WASP (Figure 4C). Directed yeast-2-hybrid analyses confirmed the Abp1/N-WASP association (data not shown).

In blot overlay analyses, the GST-Abp1 SH3 domain but not GST alone specifically interacted with purified Flag-tagged N-WASP (Figure 4D). This demonstrates a direct binding of N-WASP to the Abp1 SH3 domain.

Since N-WASP is a multi-domain protein (Figure 4E), it was indispensable to subsequently map the Abp1 interacting domain in N-WASP. All GFP-tagged N-WASP fragments containing the central proline-rich domain (PRD) were precipitated by the immobilized Abp1 SH3 domain. In contrast, GFP alone and fusion proteins lacking the proline-rich domain remained in the supernatants (Figure 4F). The association of the Abp1 SH3 domain with the isolated PRD of N-WASP (Figure 4F) demonstrates that these two domains are sufficient for the interaction.

### Abp1/N-WASP complexes exist in vivo

To determine whether endogenous Abp1 and N-WASP associate in vivo, we carried out immunoprecipitations. Anti-Abp1 antibodies effectively precipitated Abp1 from rat brain extracts (Figure 4G). Immunoblotting analyses of the precipitates with anti-N-WASP antibodies showed that N-WASP was specifically coimmunoprecipitated with Abp1 (Figure 4G). Thus, we concluded that Abp1 and N-WASP occur in the same molecular complexes in the brain.

To confirm the immunoprecipitation data and to firmly exclude that our detection of Abp1/N-WASP complexes represents a post-homogenization artifact, we reconstituted and visualized the Abp1/N-WASP interaction in intact cells by making use of a mitochondrial recruitment system [27]. N-WASP, which displays a rather diffuse cytosolic distribution and was not at all associated with mitochondria when expressed alone [27] or when cotransfected with the empty Mito-GFP vector (data not shown), adopted a mitochondrial localization pattern very similar to that of Mito-GFP-Abp1-SH3 when it was coexpressed with Mito-GFP-Abp1-SH3 (Figure 4H–J), which was effectively targeted to mitochondria (Figure S2A–C). Consistently, GFP-N-WASP encompassing a mitochondrial targeting sequence was capable to recruit both cooverexpressed full-length Abp1 (Figure S2) as well as its C-terminal part including the SH3 domain (Figure 4K–M) to mitochondria.

This in vivo reconstitution of Abp1/N-WASP interactions at cellular membranes was based on a classical PXXP-motif interaction of the Abp1 SH3 domain with N-WASP because no recruitment of the corresponding P422L and G425R SH3 domain mutant to mitochondria enriched for N-WASP (Figure 4O) was observed. Instead, Abp1 flex/SH3mut remained relatively evenly distributed within the cytosol (Figure 4N).

### Association of the SH3 domain of Abp1 with N-WASP activates N-WASP-mediated Arp2/3 complex-dependent actin polymerization

**Figure 5:**
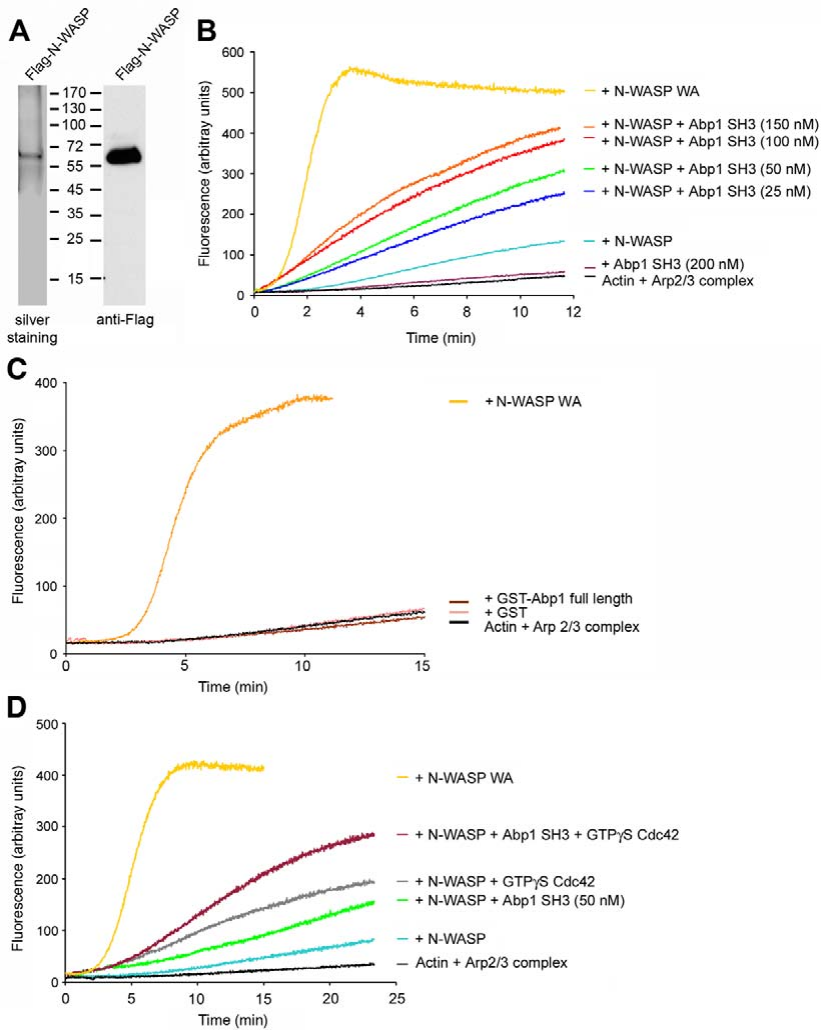
Abp1 stimulates N-WASP-mediated Arp2/3 complex-dependent actin polymerization. (A) Characterization of Flag-N-WASP that was expressed in COS-7 cells and immunoisolated from cell lysates by anti-Flag antibodies using silver staining und anti-Flag immunoblotting. (B–D) In vitro reconstitutions of actin nucleation and polymerization were analyzed by the fluorescence increase of pyrene-labeled actin. Fluorescence intensities were plotted against time. The results shown are representative of three different experiments. Polymerization induced by Arp2/3 complex and N-WASP WA (80 nM) are shown for comparison. (B) Dose-response effect of the Abp1 SH3 domain. N-WASP/Arp2/3 complex-dependent actin polymerization was examined in the absence or presence of increasing concentrations of the Abp1 SH3 domain (actin, 2 mM; pyrene-actin, 0,2 mM; Arp2/3 complex, 10 nM and Flag-tagged N-WASP, 80 nM). The Abp1 SH3 domain (200 nM) does not activate actin polymerization in absence of N-WASP. (C) Without the Arp2/3 complex activator N-WASP, full-length Abp1 (150 nM GST-Abp1) is unable to functionally interface with the Arp2/3 complex and actin kinetics are indistinguishable from that of 2 mM actin plus 10 nM Arp2/3 complex and actin plus Arp2/3 complex plus 150 nM GST, respectively. (D) Addition of GTPγS-loaded Cdc42 resulted in a partial activation of N-WASP. GTPγS-loaded Cdc42 (400 nM) acts in concert with the Abp1 SH3 domain (50 nM) in activating N-WASP.

Abp1 knock down displayed a phenotype identical to Arp2/3 complex inhibition (Figure 1) and the Abp1 SH3 domain stimulated actin polymerization in complex mixtures, such as brain extracts (Figure 2) and associates with N-WASP (Figure 4). We thus next addressed whether Abp1 may directly influence the kinetics of actin filament nucleation and polymerization stimulated by N-WASP and the Arp2/3 complex by reconstituting these processes with purified components (actin, Arp2/3 complex, N-WASP, Abp1). The kinetics of actin polymerization were measured by following the fluorescence increase of pyrene-labeled actin. To work most closely to physiological conditions, we expressed epitope-tagged full-length N-WASP in mammalian cells. Subsequent immunoisolation allowed us to obtain N-WASP in the required purity, integrity and quantities (see Figure 4D and 5A, as well as the extensive characterization of the isolated material by immunoblot analyses with antibodies against N-WASP-binding cytoskeletal components compiled in the Figure S3).

Comparable to material purified from insect cells [14], the addition of full-length N-WASP expressed in mammalian cells to a mixture of actin and the Arp2/3 complex only weakly accelerated actin polymerization (Figure 5-D), whereas the C-terminus of N-WASP, N-WASP WA, markedly activated Arp2/3 complex-induced actin polymerization in the in vitro actin polymerization assay (Figure 5B–D).

We next addressed whether the Abp1 SH3 domain would be able to release the N-WASP autoinhibition. The SH3 domain of Abp1 indeed accelerated Arp2/3 complex-induced actin polymerization in a dose-dependent manner when added in nanomolar concentrations to a reaction mixture containing N-WASP, Arp2/3 complex and actin (Figure 5B). This activation was clearly mediated through N-WASP, because the Abp1 SH3 domain had no effect in the absence of N-WASP (Figure 5B). Saturation of the Abp1 SH3 domain effect on N-WASP activation occurred at about 100-150 nM. Higher concentrations (200 nM and 400 nM) did not lead to any further increase in N-WASP activation but curves scattered around those obtained for 100 nM (data not shown).

Comparable to an SH3 domain-containing actin nucleation mixture lacking N-WASP (Figure 5B), also full-length Abp1 - although used in a concentration of 150 nM corresponding to full activation of N-WASP by the Abp1 SH3 domain (Figure 5B) - did not influence Arp2/3 complex-mediated actin dynamics (Figure 5C), while it did activate N-WASP in a manner similar to the SH3 domain (data not shown). These examinations demonstrate that Abp1 is unable to activate the Arp2/3 complex directly (Figure 5C) and are in line with the inability of mammalian Abp1 to directly associate with the Arp2/3 complex (our unpublished data).

Taken together our reconstitution experiments with purified components provide clear evidence for the involvement of Abp1 in the regulation of actin dynamics by stimulating the N-WASP/Arp2/3 complex-mediated actin polymerization reaction and indicate that Abp1 is an activator of N-WASP.

### Abp1 cooperates with Cdc42 in N-WASP activation

Cdc42 (gi|61889112) in its GTP form associates with the GTPase-binding domain (GBD) of N-WASP and thereby activates N-WASP [14], [28]. We thus analyzed whether the Abp1 SH3 domain and Cdc42 might act in concert. In order to address this, we used a fivefold excess of GTPγS-loaded Cdc42 over N-WASP. In line with the literature, addition of Cdc42 significantly accelerated N-WASP/Arp2/3 complex-mediated actin nucleation (Figure 5D). Since with the GBD there is only one Cdc42 binding site in N-WASP, the five-fold excess represents a full saturation of the system. This was confirmed experimentally by comparing the effects of 100 nM Cdc42 with those of 400 nM. As expected, the curves were similar (data not shown). We then asked whether addition of some Abp1 SH3 would further increase the activity of Cdc42-loaded N-WASP. Indeed, addition of the Abp1 SH3 domain clearly resulted in a much higher acceleration of N-WASP/Arp2/3 complex-mediated actin polymerization compared to the effects of the single N-WASP binding partners (Figure 5D). Thus, the association of Abp1 with the Arp2/3 complex-activator N-WASP does not compete with the binding of Cdc42 but both activators act in concert and in combination lead to a strong activation of N-WASP.

### N-WASP targeted to intracellular membranes elicits local actin polymerization in vivo and this effect is regulated by Abp1 and Cdc42

**Figure 6:**
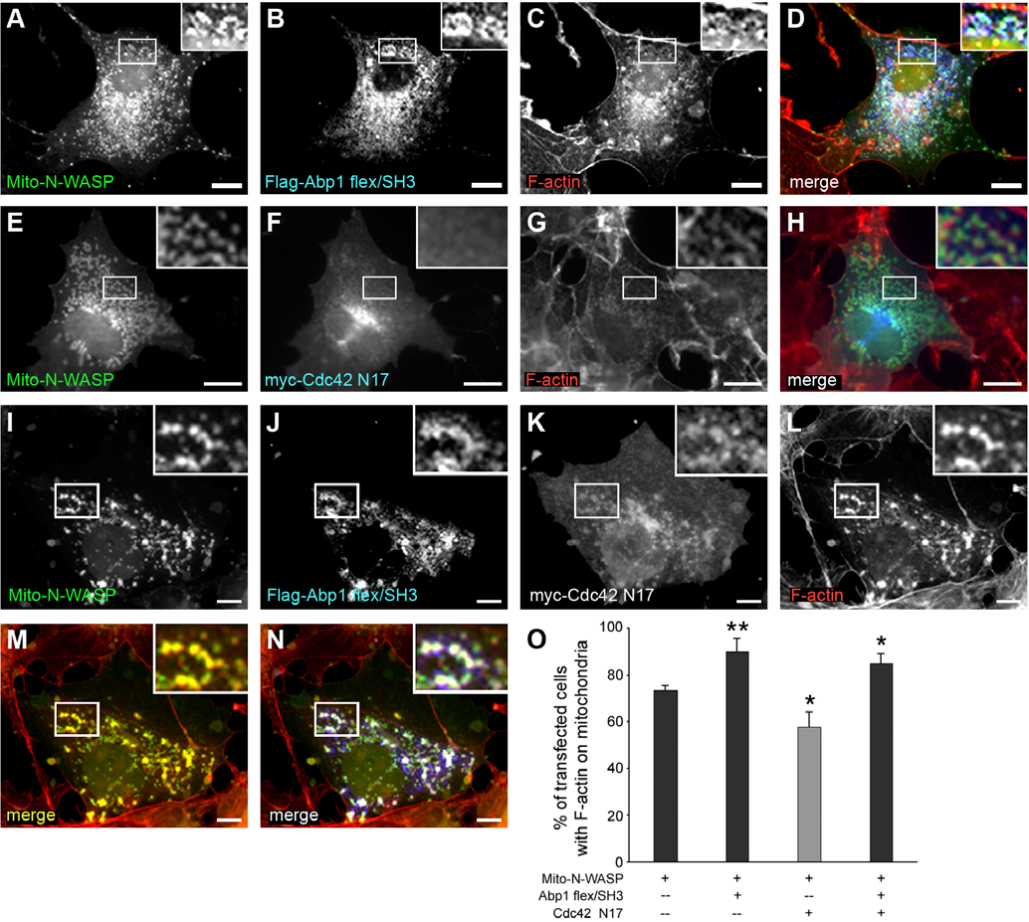
Abp1 SH3 domain association activates N-WASP in cooperation with Cdc42 in vivo. Mito-GFP-N-WASP (A) recruits Flag-Abp1 flex/SH3 (B) that was detected by anti-Abp1 immunostaining to mitochondria and such Mito-GFP-N-WASP-coated mitochondria are associated with F-actin (C). Labelling of individual panels is in the color of the respective signal in the merged images (D, H, M, N). Cooverexpression of Mito-GFP-N-WASP (E) together with dominant negative Cdc42 (Cdc42 N17) (F) caused a reduction in this mitochondrial F-actin association (G). Flag-tagged Abp1 flex/SH3 (J), cooverexpressed in addition to Mito-GFP-N-WASP (I) and Cdc42 N17 (K), restored and even increased the number of transfected cells that presented F-actin (L) on mitochondria. M corresponds to a merge of I and L. N shows I, J and L merged. (O) Quantification of the amount of cells with actin polymerized on mitochondria. Data are represented as mean±SEM (*, p<0.05; **, p<0.01). Bars = 10 µm.

While in vitro reconstitutions are state-of-the-art for studying the molecular mechanisms of Arp2/3 complex-mediated actin polymerization, we wanted to take our analyses beyond in vitro studies. We thus next designed an experimental system allowing us to address the in vivo relevance of the observed activation of N-WASP-triggered actin polymerization by Abp1 and to also study the Cdc42 cooperation in vivo. We therefore reconstituted these processes at membrane surfaces in living cells that are normally F-actin-free, the outer membranes of mitochondria. Applying a mitochondrial targeting system we observed that targeting of Mito-GFP-N-WASP (Figure S4A) but not of GFP alone (data not shown) to mitochondria resulted in a clear build-up of F-actin on their surface (Figure S4A–C). Since truncation mutants of N-WASP containing the C-terminal WA domain (Figure S4D), but for example not the PRD alone (data not shown) were also able to elicit local actin polymerization (Figure S4E), the observed actin assembly was N-WASP WA domain-mediated, i.e. Arp2/3 complex-mediated. Quantitative analyses (Figure 6O) revealed that 73±2% of the cells expressing Mito-GFP-N-WASP displayed F-actin on mitochondria indicating that N-WASP autoinhibition is partially released either through its targeting to intracellular membranes and/or by intramolecular interactions with factors that are endogenously present in COS-7 cells and are corecruited to the mitochondrial surface.

The Abp1 C-terminus, which has been proven to be necessary and sufficient for stimulating N-WASP-triggered Arp2/3 complex-dependent actin polymerization in vitro (Figure 5), is efficiently corecruited to Mito-GFP-N-WASP-decorated mitochondria and resulted in a further stimulation of local actin polymerization at mitochondrial surfaces (Figure 6A–D). This ability of the Abp1 C-terminus to activate actin filament formation mediated by N-WASP in vivo is clearly reflected in the highly significant (p<0.01) increase of transfected cells that display filamentous actin at the surface of N-WASP/Abp1-C-terminus-decorated mitochondria (Figure 6O; 90±6% in Mito-GFP-N-WASP/Abp1 flex/SH3 cotransfected cells versus 73±2% in cells transfected with Mito-GFP-N-WASP alone).

Subsequently, we applied the system to also unravel the contribution of Cdc42 in N-WASP activation in vivo. Quantification studies revealed that coexpression of dominant-negative Cdc42 N17 significantly reduced (p<0.05) the number of transfected cells exhibiting some F-actin on Mito-GFP-N-WASP-positive mitochondria (58±7%) (Figure 6O). Additionally, the amount of F-actin at mitochondria was markedly reduced (Figure 6E–H). The quantitative analysis thus significantly underestimates the suppression caused by Cdc42 N17.

The results from the in vitro actin polymerization assays suggested that the SH3 domain of Abp1 activates N-WASP-mediated actin polymerization in concert with Cdc42. If this is of in vivo relevance as well, then increasing N-WASP activation by the Abp1 SH3 domain should at least partially overcome the dominant-negative effect of Cdc42 N17. Indeed, qualitative (Figure 6I–N) and quantitative examinations (Figure 6O) showed that cooverexpression of the Abp1 C-terminus in addition to Mito-GFP-N-WASP and Cdc42 N17 restored and even increased the number of transfected cells that presented filamentous actin on mitochondria compared to cells expressing Mito-GFP-N-WASP alone. Additionally, also the amount of F-actin at Mito-GFP-N-WASP-enriched mitochondria was much higher in cells coexpressing Cdc42 N17 and the Abp1 C-terminus (Figure 6L) than in cells coexpressing Cdc42 N17 alone (Figure 6G). These experiments provide strong evidence that, also in vivo, the F-actin-binding protein Abp1 and the small GTPase Cdc42 cooperate to activate N-WASP triggered Arp2/3 complex-mediated actin polymerization.

### Neuronal morphology control mediated by N-WASP is dependent on Abp1 and Cdc42 in vivo

The enrichment of Abp1, N-WASP and the Arp2/3 complex in actin-rich growth cones of young hippocampal neurons (Figure 3) prompted us to investigate whether the Abp1-regulated N-WASP-mediated actin poly­meri­za­tion that we had observed in the in vitro reconstitution systems and upon re­con­stitution of Abp1/N-WASP complexes at intracellular membranes (Figure 5 and 6) would affect the outgrowth and morphology of neurites.

**Figure 7:**
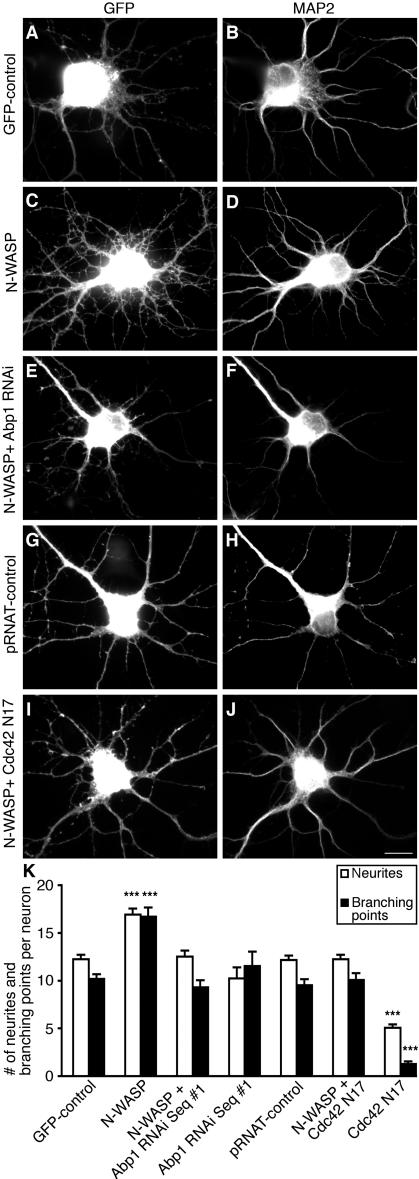
Abp1 and Cdc42 are crucial for the N-WASP-induced increase in neurites and branching points. Primary hippocampal neurons were transfected with pEGFP (GFP-control; A, B), with N-WASP (C, D), with N-WASP plus a pRNAT/mRFP-driven Abp1 RNAi construct (E, F), with pRNAT-GFP (pRNAT-control; G, H), with N-WASP in combination with myc-Cdc42 N17 (I, J), as well as with the pRNAT/mRFP-driven Abp1 RNAi construct and myc-Cdc42 N17 alone (no images shown; see K for quantitative data), respectively, at day 5 in vitro and fixed 38 h later. The number and branching of MAP2-immunopositive neurites (B, D, F, H, J) are markedly increased upon N-WASP overexpression, as also evident from quantitative analyses (K). This N-WASP effect was completely suppressed by Cdc42 N17 coexpression and reduction of Abp1 levels, respectively (K). Data are represented as mean±SEM (***, p<0.001). Bars = 10 µm.

Primary hippocampal neurons were transiently transfected with GFP (Figure 7A) and GFP-N-WASP (Figure 7C), respectively, at day 5 in culture and fixed 38 hours later. Neurons were identified and neuronal morphology was visualized by microtubule-associated protein 2 (MAP2; gi|547890) staining (Figure 7B, 7D, 7F, 7H, and 7J). Neurons transfected with GFP-N-WASP (Figure 7C) showed a prominent increase in the number of neurites extending from each neuron when compared to control cells transfected with GFP alone (Figure 7A). Additionally, the neurite network extending from N-WASP-overexpressing cells appeared highly branched (Figure 7C). Quantitative evaluations (Figure 7K) demonstrated that the N-WASP-induced increase in both the number of neurites and the number of dendritic branching points per neuron was highly significant (16.9±0.6 neurites per N-WASP-transfected cell versus 12.2±0.5 in GFP control; p<0.001; 16.7±1.0 branch points per cell versus 10.2±0.5 for GFP-control; p<0.001).

A potential critical role of Abp1 in N-WASP-mediated control of neuronal morphology was then addressed by RNAi. Cotransfection of neurons with N-WASP and an Abp1 RNAi vector clearly suppressed the effects of N-WASP overexpression (Figure 7E). The number of neurites (12.5±0.6) as well as of branching points per neuron (9.3±0.8) (Figure 7E and 7K) was significantly reduced and was indistinguishable from that of control neurons (Figure 7A and 7K). Neither Abp1 RNAi alone nor expression of the RNAi vector had significant impact on neurite number and branching, these parameters were not statistically different from control cells showing that very specifically the N-WASP overexpression phenotype was suppressed and dendrite development progresses normally (Figure 7K). Comparable to Abp1 RNAi, cooverexpression of the dominant-negative form of Cdc42, Cdc42 N17 (Figure 7I), also suppressed the N-WASP-induced morphological changes on neurite outgrowth (12.3±0.5) and branching (10.0±0.7; Figure 7K). However, overexpression of Cdc42 N17 alone already grossly affected dendritogenesis (Figure 7K). Taken together, these data clearly show that Abp1 and Cdc42 modulate the activity of the Arp2/3 complex activator N-WASP in neurons and that they are essential for the rearrangements of the cytoskeleton that are induced by N-WASP and control cellular morphology.

### Reduction or functional inhibition of N-WASP caused an axonal development phenotype resembling that induced by Abp1 or Arp2/3 complex deficiency

**Figure 8:**
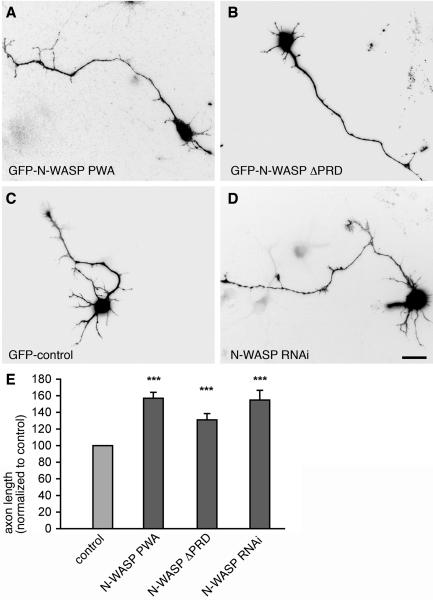
N-WASP mutants deficient for Abp1 or Cdc42 binding and N-WASP RNAi cause increased axon lengths. Primary hippocampal neurons overexpressing GFP-N-WASP PWA (A) and GFP-N-WASP ?PRD (B) show significantly (***, p<0.001) elongated axons when compared to control neurons expressing GFP alone (C). Similarly, N-WASP RNAi (D) leads to elongated axons in comparison to control. E, quantitative evaluations of axon length. Data were normalized to control and are represented as mean (in percent)±SEM. Bar = 10 µm.

We observed strong deficites in axon development when we reduced the availability of the Arp2/3 complex or Abp1 (Figure 1) and revealed that Abp1 interacts directly with the Arp2/3 complex activator N-WASP. It was therefore of particular importance to next address the function of Abp1/N-WASP complexes in axonal development. In order to interfere with such complexes, we deleted the binding interfaces for Abp1 and Cdc42 on N-WASP. These manipulations should mimic the effects of Abp1 knock down and of Arp2/3 complex inhibition, respectively, on axon length in young neurons because such N-WASP mutants deficient for Abp1 and Cdc42 binding can be expected to compete with endogenous N-WASP and thereby disturb its functions. Indeed, both hippocampal neurons overexpressing GFP-N-WASP PWA (Figure 8A), i. e. a mutant that has no means to bind to Cdc42, as well as neurons overexpressing GFP-N-WASP ΔPRD (Figure 8B), i. e. a mutant lacking the Abp1 binding interface, exhibited significantly (p<0.001) elongated axons when compared to control cells (Figure 8C). These phenotypes were further validated by quantitative analyses (Figure 8E). Overexpression of wild-type N-WASP analyzed in additional control experiments did not lead to the highly significant increase in axon length observed with the mutants (data not shown). This excludes the possibility that the phenotypes observed merely represent N-WASP overexpression-related effects. Additional experiments interfering with the availibility of N-WASP in toto proved that the axon developmental defects observed by preventing Cdc42 and Abp1 association were indeed specifically reflecting disturbances in N-WASP function. N-WASP RNAi (achieved according to Yamaguchi et al. [29]) caused overshooting axon growth as well (Figure 8D). The phenotypes of interfering with N-WASP functions by the introduction of mutants incapable to bind to Abp1 or Cdc42 are thus identical to those obtained by simply reducing N-WASP as a whole (Figure 8E). Thus, cooperative action of Abp1 and Cdc42 appears to be indispensable for controlling N-WASP/Arp2/3 complex-mediated actin polymerization required for correct formation of axons and thereby for proper neuronal morphogenesis and neuronal network formation.

## Discussion

The integrity of the actin cytoskeleton and its ability to reorganize rapidly in response to external stimuli and internal cues is indispensable for changes in cell shape and motility but also for a variety of intracellular functions. These features of the actin cytoskeleton are regulated by a diverse array of actin-binding proteins, which are thereby modulators of cellular dynamics and key components of signaling processes. We here report that the mammalian F-actin-binding protein Abp1 plays an indispensable role in Arp2/3 complex-mediated actin polymerization controlled by the Arp2/3 complex activator N-WASP. We demonstrate that Abp1 directly interacts with N-WASP and that Abp1/N-WASP complexes exist both in brain extracts and in intact cells. Our reconstitutions of actin polymerization with purified components in vitro clearly demonstrate that Abp1 stimulates Arp2/3 complex-mediated actin filament assembly via activation of the catalytic activator N-WASP.

The moderately related counterpart of Abp1 in *S. cerevisiae*, Abp1p, has been demonstrated to interface directly with the Arp2/3 complex [26]. In vitro experiments with yeast Abp1p and Las17p (gi|1420437), the ancestor of the WASP superfamily of proteins in *S. cerevisiae,* demonstrated that these proteins compete with each other for Arp2/3 complex binding and that Abp1p presence drastically reduced Las17p/Arp2/3 complex-mediated actin polymerization. In yeast, Abp1p was thus rather considered as an inhibitor of Arp2/3 complex-mediated actin polymerization than as an activator because Las17p-mediated actin nucleation seems to be more powerful than Abp1-mediated actin nucleation [30]–[31]. This, however, seems to be a specialty of yeast. First, the acidic stretches in the yeast protein mediating the Arp2/3 complex interaction are not conserved in Abp1 proteins of higher eukaryotes. Consistently, we found that mammalian Abp1 does neither associate with nor activate the Arp2/3 complex directly. Second, both our in vitro and our in vivo experiments clearly reveal that Abp1 and N-WASP work together in higher eukaryotes in promoting actin polymerization and that the ability of mammalian Abp1 to interface functionally with the Arp2/3 complex relies on SH3 domain-mediated interactions with Arp2/3 complex activators. The indirect activation of the actin polymerization machinery in mammalian cells by Abp1 via N-WASP might – from an evolutionary point of view – represent an improvement, because N-WASP is a multidomain protein whose activity can be modulated through a variety of signaling pathways [10]–[11] and this significantly extends the possibilities for fine-control of actin filament nucleation. Indeed, both the in vitro reconstitution of actin polymerization and the reconstitution of Abp1/N-WASP complexes at membranes in vivo clearly revealed that the Abp1 SH3 domain, i.e. the binding interface for N-WASP, acts in concert with the small Rho-type GTPase Cdc42 to regulate N-WASP-stimulated Arp2/3 complex-dependent actin polymerization. Such an involvement of multiple signals in N-WASP activation is consistent with the idea that WASP family proteins represent important integration points in signaling pathways towards the actin cytoskeleton.

Strikingly, the degree of cooperative action between Cdc42 and proteins reported to associate with the PRD of N-WASP shows major differences in in vitro reconstitutions. Whereas Grb2 (gi|2498425) has been reported to act synergistically with Cdc42 in relieving the autoinhibited conformation of N-WASP and thereby in enhancing N-WASP-mediated actin polymerization [32] and Abi1 (gi|50400218) cooperates with Cdc42 in enhancing N-WASP activity [33] similar to Abp1 [this study], the effects of Nck-1 (gi|34328187) and Cdc42 were found to be less than additive [34] and WISH-stimulated (gi|49258190) N-WASP-induced Arp2/3 complex activation was not at all increased by the addition of active Cdc42 [35]. It is, however, largely unclear whether and how these proteins cooperate with Cdc42 in N-WASP control in vivo.

Our examinations of Abp1 functions clearly demonstrate that Abp1 can activate N-WASP and cooperates with Cdc42 not only in vitro but also in vivo. The data from the in vivo reconstitutions of N-WASP/Arp2/3 complex-mediated actin polymerization at intracellular membranes are hereby very well in line with the finding that, in neurons, the N-WASP-induced increases of neurites and of neuritic branch points were very effectively suppressed by both Abp1 RNAi and dominant-negative Cdc42 and is thus dependent on both Abp1 and Cdc42. The fact that Abp1 RNAi alone had no effect on dendritogenesis is important to note because this demonstrates that specifically the effects of the excess of Abp1/N-WASP complexes are blocked by reducing Abp1 and excludes that putative indirect effects, which are unrelated to N-WASP, account for the suppression of the N-WASP overexpression phenotype.

A special feature of Abp1 as N-WASP activator is that in addition to its N-WASP interacting domain it has two N-terminal F-actin binding modules. Abp1/N-WASP/Arp2/3 complexes can thus associate with F-actin via both the side binding activity of the Arp2/3 complex [36] and via Abp1's ability to bind actin fibers [17]. This may be an important mechanism for the generation of new sites of nucleation on actin fibers and thereby promote branched actin superstructures. Since in vivo, Abp1 specifically associates in a signal-responsive manner with highly dynamic F-actin newly generated by Arp2/3 complex activity [17], assembly of Abp1/N-WASP/Arp2/3 complexes at such sites will act as a feed-forward mechanism promoting branched structures. Such an effective generation of highly branched and complex F-actin networks is especially important for the formation of the dynamic actin structures at the leading edge of lamellipodia, of short-lived actin structures occurring at sites of endocytosis and of the actin networks in neuronal growth cones that are important for their organization and neuronal pathfinding. Consistently, all these structures are marked by both Arp2/3 complex and Abp1 accumulation [17–18, 23, 37–38; this study].

The colocalization of Abp1, N-WASP and Arp2/3 complex in growth cones we observed suggested that all three components mediate actin dynamics in neurite outgrowth and differentiation. The identical phenotypes of Abp1 and Arp3 knock down as well as Arp2/3 complex inhibition on axon outgrowth strongly support this view. The finding that suppression of Abp1 and Arp2/3 complex-mediated actin nucleation promotes axon outgrowth hereby seems somewhat counter-intuitive considering the well-established role of the Arp2/3 complex in actin nucleation and its pivotal role in creating the branched actin structures extending lamellipodia during cell migration [9]–[10]. Our quantitative data for Arp2/3 inhibition are, however, very well in line with the measurements of Strasser et al. [23] and are corroborated by the effects we observed upon Arp3 RNAi.

Two conclusions can be drawn from these findings, first, at least in neurons, there must be considerable functional redundancy of the Arp2/3 complex with other, not yet identified actin nucleators that help to ensure that neurites are formed, elongated and branched properly to give rise to polarized cells that can form functional neuronal networks. It will be of extreme importance to identify these factors, as their discovery and characterization will lead to a deeper understanding in neuronal morphology control and in mechanisms of actin nucleation in general. Second, in axonal growth cones, Arp2/3 complex functions seem to interfere to some extend with the mere extension of the growth cone. The work of Strasser et al. [23] suggested that Arp2/3 complex-mediated organization of branched filaments and three-dimensional networks residing in the center of growth cones may be important for growth cone translocation and guidance because these actin networks also functionally interface with the microtubule cytoskeleton. In this context, it is conceivable that inhibiting the formation of these higher order structures by knocking down Abp1 and Arp3, respectively, or by inhibiting the Arp2/3 complex may further promote the rapid extension of the mostly unbranched and parallel actin fibers in the periphery of axonal growth cones that are formed independently of Arp2/3 complex functions and drive growth cone extension.

Importantly, our data do not only show that Abp1 and the Arp2/3 complex function in the same cellular process but that also the molecular link between Abp1 and Arp2/3 that we discovered, N-WASP, does so. Expressing either an N-WASP mutant that is able to interact with Cdc42 but lacks the Abp1-binding PRD or expressing an N-WASP mutant that can undergo Abp1 binding but lacks the Cdc42 binding N-terminal part also caused phenotypes similar to that of Arp2/3 complex inhibition, Arp3 RNAi or Abp1 RNAi. The same is true for reducing the availability of N-WASP as a whole by RNAi. Together, these data strongly suggest that Abp1, N-WASP and the Arp2/3 complex work together in one complex, as demonstrated by our biochemical analyses, and that Abp1 is a crucial component in N-WASP/Arp2/3 complex-mediated cytoskeletal functions.

Our N-WASP RNAi data are well in line with results very recently reported by Kakimoto et al. [39]. This study compared the effects of N-WASP and Toca-1 (gi|87299584) RNAi in hippocampal neurons and revealed that in contrast to interfering with N-WASP, knock down of the N-WASP binding and activating protein Toca-1 failed to cause any effects on axon elongation. It can thus be concluded that Toca-1, although it binds N-WASP and releases its autoinhibition in vitro, regulates neuronal morphology aspects different from N-WASP. The study by Kakimoto et al. [39] thereby highlights that it is of extreme importance to substantiate in vitro data for N-WASP autoinhibition release by N-WASP-binding proteins with studies in vivo and with examinations that address the functional cooperativity of N-WASP and a given N-WASP binding protein in physiological processes. Combining protein interaction studies, in vitro and in vivo reconstitutions with overexpression, mutational and protein knock down studies in developing neurons our data clearly show that Abp1 is an important component in N-WASP/Arp2/3 complex-mediated actin dynamics in neuronal morphology control and network formation.

In addition to control of neuronal morphology, N-WASP-mediated Arp2/3 complex-dependent actin polymerization controlled by Abp1 might play a role during endocytic vesicle formation, a process supported by transient, local actin nucleation [4]–[5]. Both Abp1 [18]–[20] and N-WASP [27], [33], [40] have been shown to be important components for endocytic internalization. Since the dominant-negative effect of N-WASP on receptor-mediated endocytosis is exclusively dependent on the PRD [27], i.e. the binding interface of Abp1, Abp1 might also integrate N-WASP functions into endocytosis. Further studies are required to reveal the mechanistic functions of Abp1/N-WASP complexes in this process.

In mature neurons, a functional crosstalk between the cytoskeletal and membrane trafficking machineries might be of particular importance in nerve terminals to ensure the high speed, efficiency and accuracy of vesicle formation and recycling – important for both synaptic transmission and plasticity [7]. In line with this hypothesis, we observed N-WASP and Abp1 as well as the Arp2/3 complex at sites of the neuronal network in mature neurons that are likely to represent synapses. Abp1 localizes to both the pre- and the postsynaptic compartment and binds specifically to cytomatrix components involved in the organization and orchestration of subsynaptic structure and functions at both sites of the synaptic cleft [21]–[22]. Together with its F-actin binding capabilities and its ability to induce Arp2/3 complex-mediated actin polymerization, Abp1 therefore integrates essential features for a key organizing element for the establishment and reorganization of neuronal networks and synaptic contacts.

## Materials and Methods

### DNA Constructs and Recombinant Proteins

The expression and purification of the Abp1 GST fusion proteins used in this study were described previously [17], [18], [41]. For the expression of myc- and Flag-tagged Abp1 flex/SH3 domain, Abp1 (282–433) was subcloned into pCMV-Tag2 and pCMV-Tag3, respectively (Stratagene). In order to generate an RNAi-resistant Abp1 mutant several silent mutations (ATTCCGGAAAAG; mutations underlined) were introduced by PCR into the Abp1 cDNA site targeted by RNAi sequence #1. The PCR product was then cloned into pCMV-Tag2 and verified by DNA sequencing.

Mammalian expression constructs encoding GFP-N-WASP and GFP-N-WASP ΔPRD as well as HA-tagged N-WASP were described in [27]. Additionally, GST- and GFP-tagged N-WASP WA as well as GST-tagged N-WASP PWA were generated by subcloning. GFP-N-WASP CA (464–501) was generated by PCR and cloning into pEGFP-C1. Full-length N-WASP was subcloned into pCMV-Tag2 for the expression of Flag-tagged N-WASP. For in vivo recruitment experiments, full-length N-WASP, full-length Abp1 and the SH3 domain of Abp1 were inserted into a derivative of our mitochondrial-targeting vector [42]. A plasmid encoding myc-Cdc42 N17 was generously provided by A. Hall (University College London, London, United Kingdom).

To generate RNAi constructs directed against rat Abp1 the following oligonucleotides were annealed and subcloned into pRNAT H1.1-GFP (GenScript), Abp1 RNAi sequence #1: 5-GATCCCAGCGGGAAGGTGATGTACTTGATATCCGGTACATCACCTTCCCGCTGTTTTTTA-3 and 5-AGCTTAAAAAACAGCGGGAAGGTGATGTACCGGATATCAAGTACATCACCTTCCCGCTGG-3, Abp1 RNAi sequence #2: 5-GATCCGGTGATGTACGCCTTCTGCTTGATATCCGGCAGAAGGCGTACATCACCTTTTTTA-3 and 5-AGCTTAAAAAAGGTGATGTACGCCTTCTGCCGGATATCAAGCAGAAGGCGTACATCACCG-3. Residues mutated for generation of an RNAi-resistant Abp1 are underlined (in RNA sequence #1). A non-silencing RNAi control was generated with the following oligos: GATCCAATTCTCCGAACGTGTCACGTTTGATATCCGACGTGACACGTTCGGAGAATTTTTTTTA-3 and 5- AGCTTAAAAAAAATTCTCCGAACGTGTCACGTCGGATATCAAACGTGACACGTTCGGAGAATTG -3. RNAi tools against N-WASP and the Arp2/3 complex component Arp3 were generated by annealing and subcloning oligonucleotides corresponding to the siRNAs established by [29] and [24], respectively, into pRNAT H1.1-GFP. All sequences used were checked for specificity by BLAST searches against genome databases. A pRNAT-driven Abp1 RNAi construct coexpressing monomeric red fluorescent protein (mRFP) was generated by replacing GFP with mRFP.

For the actin polymerization assays, GST-fusion proteins of N-WASP WA and N-WASP PWA were expressed and purified according to methods described previously [17], [41]. Similarly, the SH3 domain of Abp1 was expressed as GST-fusion protein, purified on glutathione sepharose 4B (Amersham Biosciences), cleaved from the GST-tag using thrombin (Sigma) and purified by gel filtration.

For expression and purification of Flag-tagged N-WASP, 16 bottles (75 cm^2^) of COS-7 cells were transiently transfected according to the DEAE-Dextran procedure (Promega Transfection Guide, 1999) and butyrated 24 h after transfection, as described [43]. After 36–48 h the cells were lysed for 20 min in 0.5 ml lyses buffer (10 mM HEPES, 1 mM EGTA, 0.1 mM MgCl_2_, 150 mM NaCl, 1% Triton X-100, pH 7.5) supplemented with an EDTA-free protease inhibitor cocktail (Roche) and spun for 20 min at 14,000 *g*. The supernatant was incubated overnight with anti-Flag antibodies covalently attached to protein G sepharose (see below). After several washes with TBS (10 mM TRIS/HCl, 150 mM NaCl, pH 7.4) Flag-N-WASP was eluted by incubation with 1.5 ml of TBS containing 100 µg/ml Flag-peptide (Sigma) for 12 h and dialyzed against PBS.

For antibody coating, 300 µl of protein G sepharose 4 Fast Flow (Amersham Biosciences) were incubated with 1 ml of 0.24 mg/ml anti-Flag antibodies in PBS containing 5% BSA for 5 h. After several washes with HEPES buffer (10 mM HEPES, 1 mM EGTA, 0.1 mM MgCl_2_, pH 7.4) the resin was incubated for 0.5 h with 0.4 ml of 10 mM *N*-(3-dimethylaminopropyl)-*N*′-ethylcarbodiimide hydrochloride (Sigma) in HEPES buffer. Beads were washed twice with HEPES buffer and once with lyses buffer prior to incubation with cell extracts. All steps of the lysis and purification procedure were carried out at 4°C.

### Antibodies

Rabbit and guinea pig anti-Abp1 antibodies, guinea pig anti-N-WASP (P337) antibodies, and rabbit anti-GST antibodies were described previously [17], [25], [27], [41]. Rabbit anti-Arp3 antibodies were kindly provided by M. D. Welch (University of California, Berkeley, CA, USA).

Monoclonal anti-MAP2, anti-actin and anti-Flag (M2) antibodies were from Sigma, monoclonal anti-GFP (B34) and anti-myc (9E10) antibodies were from Babco. Rabbit anti-profilin antibodies were from Cytoskeleton. Rabbit anti-myc (A-14) antibodies were from Santa Cruz Biotechnology, Inc., rabbit anti-GFP antibodies were from Abcam.

Secondary antibodies used in this study included Alexa Fluor® 350, 488 and 568 goat anti-mouse, Alexa Fluor® 350, 568 and 647 goat anti-rabbit, and Alexa Fluor® 647 goat anti-guinea pig antibodies from Molecular Probes; goat anti-guinea pig FITC antibodies from ICN Biomedicals; and goat anti-rabbit Cy5 and donkey anti-mouse Cy5 antibodies from Dianova.

Peroxidase-conjugated donkey anti-rabbit and goat anti-mouse antibodies were from Jackson ImmunoResearch Laboratories and peroxidase-conjugated rabbit anti-guinea pig antibodies were from DakoCytomation.

### Blot Overlay and Coprecipitation Assays

Blot overlay experiments were performed according to [41]. Coprecipitations were essentially performed as described [18], [22], [27], [43].

### Actin polymerization bead assay

The actin polymerization bead assay was essentially designed according to [44]. GST-fusion proteins were immobilized on glutathione sepharose 4B beads (Amersham Biosciences). To generate brain extracts, frozen rat brains were diced and homogenized 1∶1 (w/v) in RBC buffer (10 mM HEPES, 1 mM ATP, 0.5 mM DTT, pH 7.0, supplemented with an EDTA-free protease inhibitor cocktail (Roche)) with a Potter S homogenizer (B. Braun Biotech International) at 900 rpm. Insoluble material was removed by centrifugation for 30 min at 100,000 *g*. 20 µl of the high speed supernatant was supplemented 1∶10 with 150 mM creatine phosphate, 20 mM ATP and 20 mM MgCl_2_. Additionally, 2.5 µl of 10 µM Alexa Fluor® 568 G-actin (Molecular Probes) in 50% glycerol were added. The polymerization reaction was initiated by adding coated beads. Samples were incubated on ice for 5 min and analyzed by fluorescence microscopy.

### Pyrene-actin polymerization assay

The pyrene-actin polymerization assay was designed according to [45]. Reaction mixtures contained 10 nM Arp2/3 complex (Cytoskeleton), 80 nM Flag-N-WASP and additional proteins in 104.5 μl buffer (10 mM HEPES, pH 7.6, 100 mM KCl, 1 mM MgCl_2_, 0.1 mM EDTA, 1 mM DTT) and were preincubated for 5 min at RT. The reaction was initiated by adding 5.5 µl of a mixture of 40 µM unlabeled G-actin, 4 µM pyrene-labeled G-actin and 4 mM ATP (pH 7.0). The change in fluorescence was measured at 407 nm in a fluorescence spectrometer (excitation at 365 nm; Fluoromax 3, Jobin Yvon, Horiba Group). Cdc42 (Cytoskeleton) was preincubated with 20 mM EDTA and 40 µM GTPγS for 15 min at 30°C. The reaction was stopped by adding 20 mM MgCl_2_.

### Coimmunoprecipitation from Rat Brain Extracts

Immunoprecipitations were performed with affinity-purified rabbit anti-Abp1 antibodies or unrelated rabbit IgG (Santa Cruz) from rat brain extracts (1 mg each) in the presence of 100 mM NaCl as described [22].

### Cell Culture and Immunofluorescence Microscopy

HEK293 and COS-7 cells were maintained and transfected as described [18]. Primary hippocampal cultures were prepared and processed for immunofluorescence as described previously [18]. Transfections of neurons were performed at day 2 and 5 in vitro, respectively, according to the instructions of the manufacturer, using 1 µg of Lipofectamine 2000 (Invitrogen) and 1 µg DNA per well of a 24-well plate. Neurons were fixed in 4% PFA in PBS (pH 7.4) for 8 min at room temperature 24 h and 38 h after transfection, respectively.

For mitochondrial staining, cells were incubated with MitoTracker® Red CMXRos or Deep Red 633 (Molecular Probes) as described [27]. For F-actin staining, fixed cells were incubated with Texas Red®-X, Alexa Fluor® 488 or Alexa Fluor® 568 phalloidin from Molecular Probes.

Images were recorded digitally using a Zeiss Axioplan 2 microscope or a Zeiss Axio-Imager.D1 both equipped with a CCD camera 2.1.1 from Diagnostic Instruments and processed in MetaVue or Spot Software and Adobe Photoshop.

To assess the effect of the reconstitution of N-WASP/Abp1 complexes at mitochondrial membranes, all transfected cells on several coverslips were identified by GFP fluorescence or by immunolabeling and scored for phalloidin staining on the mitochondria. The presence of F-actin on mitochondria was only analyzed in cells with Mito-GFP-N-WASP correctly targeted to mitochondria and, if contransfected with Flag-Abp1 flex/SH3, with Flag-Abp1 flex/SH3 corecruited. About 180–500 cells were scored for each group. Results from at least 3 independent experiments were averaged and subjected to statistical significance calculations using the two-tailed Student test.

Morphometric measurements of transiently transfected hippocampal neurons were performed with the aid of the NIH Image Software (ImageJ). Each experiment was repeated at least three times with independent neuronal preparations. Neurons were identified by anti-MAP2 staining and sampled randomly for morphometric analyses. The number of neurites protruding from the cell body and the number of neuritic branching points from 29–46 neurons for each condition were counted and measured at day 6 in culture. Axon and dendrite lengths as well as neurite numbers were determined in 46–67 neurons each (at day 3 in culture). Axons of immature neurons were defined as longest neurite at that time. Statistical analysis was performed using the two-tailed Student test.

## Supporting Information

Figure S1 RNAi-based reduction of Abp1 expression levels. (A–D) Primary hippocampal neurons were transfected at day 5 in culture with a vector encoding for GFP and small interfering RNAs complementary to the Abp1 message under two different promotors. Neuronal cells were identified by anti-MAP2 immunostaining (C). Neurons transfected with pRNAT-driven Abp1 RNAi sequence #1 are marked by GFP expression (arrow; A) and showed a significant reduction in the anti-Abp1 immunoreactivity (B). Labelling of images reflects the color of the fluorescence signal in the merged image (D; colocalization appears yellow). (E) Quantitative analysis of the anti-Abp1 immunoreactivity of 50 neurons transfected with pRNAT-driven Abp1 RNAi construct (marked by GFP coexpresion) demonstrates that both Abp1 RNAi sequences tested result in an almost 60% reduction of the anti-Abp1 immuno-fluorescence intensity when compared to the pRNAT control. Data are represented as mean±SEM. Bar = 20 µm.
(5.27 MB TIF)

Figure S2 Reconstitution of Abp1/N-WASP complexes at intracellular membranes. N-WASP and Abp1 SH3 domain fusion proteins encompassing a mitochondrial targeting sequence are recruited efficiently to mitochondrial membranes. COS-7 cells were transfected with Mito-GFP-Abp1 SH3 domain (B) and with Mito-GFP-N-WASP (E), respectively. Both Mito-GFP-Abp1 SH3 domain (B) and Mito-GFP-N-WASP (E) were targeted successfully to mitochondria, which were stained with MitoTracker® (A, D). Labelling of images reflects the color of the fluorescence signal in the merged images (C, F, I; colocalization appears yellow). Inserts represent higher magnifications of the boxed areas. Mito-GFP-N-WASP (H) is able to corecruit myc-tagged Abp1 full-length (G) in vivo, as evident by the obtained colocalization on mitochondria (I). Bars (A–F) = 15 µm; bars (G–I) = 10 µm.
(5.24 MB TIF)

Figure S3 Characterization of immunoisolated Flag-N-WASP by immunoblotting. Flag-tagged N-WASP immunoisolated from COS-7 cells was analyzed further by immunoblotting with different antibodies. Anti-Flag and anti-N-WASP incubations show that the material is intact and of correct size (compare immunosignal of endogenous N-WASP in rat brain extracts (RBC) obtained with anti-N-WASP antibodies). Further analyses demonstrated that the immunoisolation protocol employed is suitable to yield N-WASP material free of direct N-WASP binding partners, such as actin, the Arp2/3 complex component Arp3, profilin (gi|6755040) and Abp1. 50 µg RBC was loaded as positive control for each antibody.
(6.39 MB TIF)

Figure S4 Mito-N-WASP-induced actin polymerization on mitochondrial membranes is mediated by the Arp2/3 complex-interacting C-terminal WA domain COS-7 cells transfected with mitochondrially targeted full-length N-WASP (A) or N-WASP WA (D) showed presence of F-actin polymerized specifically at places of N-WASP targeting, as seen in the Alexa Fluor® 568 phalloidin staining (B, E). Labelling of images reflects the color of the fluorescence signal in the merged images (C and F; colocalization appears yellow). (G) Schematic representation of the parts of the N-WASP protein that trigger Arp2/3 complex mediated actin polymerization at mitochondrial membranes. Bars = 10 µm.
(4.32 MB TIF)

## References

[1] Pollard TD, Borisy GG (2003). Cellular motility driven by assembly and disassembly of actin filaments.. Cell.

[2] Suetsugu S, Takenawa T (2003). Regulation of cortical actin networks in cell migration.. Int Rev Cytol.

[3] Qualmann B, Mellor H (2003). Regulation of endocytic traffic by Rho GTPases.

[4] Qualmann B, Kessels MM, Kelly RB (2000). Molecular links between endocytosis and the actin cytoskeleton.. J Cell Biol.

[5] Qualmann B, Kessels MM (2002). Endocytosis and the cytoskeleton.. Int Rev Cytol.

[6] Dent EW, Gertler FB (2003). Cytoskeletal dynamics and transport in growth cone motility and axon guidance.. Neuron.

[7] Gundelfinger ED, Kessels MM, Qualmann B (2003). Temporal and spatial coordination of exocytosis and endocytosis.. Nat Rev Mol Cell Biol.

[8] Dillon C, Goda Y (2005). The actin cytoskeleton: integrating form and function at the synapse.. Annu Rev Neurosci.

[9] Welch MD, Mullins RD (2002). Cellular control of actin nucleation. Annu Rev Cell Dev Biol 18: 247–288.. Biochem J.

[10] Millard TH, Sharp SJ, Machesky LM (2004). Signalling to actin assembly via the WASP (Wiskott-Aldrich syndrome protein)-family proteins and the Arp2/3 complex.. Biochem J.

[11] Takenawa T, Miki H (2001). WASP and WAVE family proteins: key molecules for rapid rearrangement of cortical actin filaments and cell movement.. J Cell Sci.

[12] Miki H, Miura K, Takenawa T (1996). N-WASP, a novel actin-depolymerizing protein, regulates the cortical cytoskeletal rearrangement in a PIP2-dependent manner downstream of tyrosine kinases.. EMBO J.

[13] Kim AS, Kakalis LT, Abdul-Manan N, Liu GA, Rosen MK (2000). Autoinhibition and activation mechanisms of the Wiskott-Aldrich syndrome protein.. Nature.

[14] Rohatgi R, Ma L, Miki H, Lopez M, Kirchhausen T et al (1999). The interaction between N-WASP and the Arp2/3 complex links Cdc42-dependent signals to actin assembly.. Cell.

[15] Higgs HN, Pollard TD (2001). Regulation of actin filament network formation through ARP2/3 complex: activation by a diverse array of proteins.. Annu Rev Biochem.

[16] Stradal TE, Rottner K, Disanza A, Confalonieri S, Innocenti M (2004). Regulation of actin dynamics by WASP and WAVE family proteins.. Trends Cell Biol.

[17] Kessels MM, Engqvist-Goldstein ÅEY, Drubin DG (2000). Association of mouse actin-binding protein 1 (mAbp1/SH3P7), a src kinase target, with dynamic regions of the cortical actin cytoskeleton in response to Rac1 activation.. Mol Biol Cell.

[18] Kessels MM, Engqvist-Goldstein ÅEY, Drubin DG, Qualmann B (2001). Mammalian Abp1, a signal-responsive F-actin-binding protein, links the actin cytoskeleton to endocytosis via the GTPase dynamin.. J Cell Biol.

[19] Mise-Omata S, Montagne B, Deckert M, Wienands J, Acuto O (2003). Mammalian actin binding protein 1 is essential for endocytosis but not lamellipodia formation: functional analysis by RNA interference.. Biochem Biophys Res Commun.

[20] Connert S, Wienand S, Thiel C, Krikunova M, Glyvuk N (2006). SH3P7/mAbp1 deficiency leads to tissue and behavioral abnormalities and impaired vesicle transport.. EMBO J.

[21] Fenster SD, Kessels MM, Qualmann B, Chung WJ, Nash J (2003). Interactions between Piccolo and the actin/dynamin-binding protein Abp1 link vesicle endocytosis to presynaptic active zones.. J Biol Chem.

[22] Qualmann B, Boeckers T, Jeromin M, Gundelfinger ED, Kessels MM (2004). Linkage of the actin cytoskeleton to the postsynaptic density via direct interactions of Abp1 with the ProSAP/Shank family.. J Neurosci.

[23] Strasser GA, Rahim NA, VanderWaal KE, Gertler FB, Lanier LM (2004). Arp2/3 is a negative regulator of growth cone translocation.. Neuron.

[24] Steffen A, Faix J, Resch GP, Linkner J, Wehland J, Small JV (2006). Filopodia formation in the absence of functional WAVE- and Arp2/3-complexes.. Mol Biol Cell.

[25] Fucini RV, Chen J-L, Sharma C, Kessels MM, Stamnes M (2002). Golgi vesicle proteins are linked to the assembly of an actin complex defined by mAbp1.. Mol Biol Cell.

[26] Goode BL, Rodal AA, Barnes G, Drubin DG (2001). Activation of the Arp2/3 complex by the actin filament binding protein Abp1p.. J Cell Biol.

[27] Kessels MM, Qualmann B (2002). Syndapins integrate N-WASP in receptor-mediated endocytosis.. EMBO J.

[28] Miki H, Sasaki T, Takai Y, Takenawa T (1998). Induction of filopodium formation by a WASP-related actin-depolymerizing protein N-WASP.. Nature.

[29] Yamaguchi H, Lorenz M, Kempiak S, Sarmiento C, Coniglio S (2005). Molecular mechanisms of invadopodium formation: the role of the N-WASP-Arp2/3 complex pathway and cofilin.. J Cell Biol.

[30] D'Agostino JL, Goode BL (2005). Dissection of Arp2/3 complex actin nucleation mechanism and distinct roles for its nucleation-promoting factors in Saccharomyces cerevisiae.. Genetics.

[31] Kaksonen M, Toret CP, Drubin DG (2005). A modular design for the clathrin- and actin-mediated endocytosis machinery.. Cell.

[32] Carlier MF, Nioche P, Broutin-L'Hermite I, Boujemaa R, Le Clainche C (2000). GRB2 links signaling to actin assembly by enhancing interaction of neural Wiskott-Aldrich syndrome protein (N-WASp) with actin-related protein (ARP2/3) complex.. J Biol Chem.

[33] Innocenti M, Gerboth S, Rottner K, Lai FP, Hertzog M (2005). Abi1 regulates the activity of N-WASP and WAVE in distinct actin-based processes.. Nat Cell Biol.

[34] Rohatgi R, Nollau P, Ho HY, Kirschner MW, Mayer BJ (2001). Nck and phosphatidylinositol 4,5-bisphosphate synergistically activate actin polymerization through the N-WASP-Arp2/3 pathway.. J Biol Chem.

[35] Fukuoka M, Suetsugu S, Miki H, Fukami K, Endo T (2001). A novel neural Wiskott-Aldrich syndrome protein (N-WASP) binding protein, WISH, induces Arp2/3 complex activation independent of Cdc42.. J Cell Biol.

[36] Mullins RD, Stafford WF, Pollard TD (1997). Structure, subunit topology, and actin-binding activity of the Arp2/3 complex from Acanthamoeba.. J Cell Biol.

[37] Welch MD, DePace AH, Verma S, Iwamatsu A, Mitchison TJ (1997). The human Arp2/3 complex is composed of evolutionarily conserved subunits and is localized to cellular regions of dynamic actin filament assembly.. J Cell Biol.

[38] Merrifield CJ, Qualmann B, Kessels MM, Almers W (2004). Neural Wiskott Aldrich Syndrome Protein (N-WASP) and the Arp2/3 complex are recruited to sites of clathrin-mediated endocytosis in cultured fibroblasts.. Eur J Cell Biol.

[39] Kakimoto T, Katoh H, Negishi M (2006). Regulation of neuronal morphology by Toca-1, an F-BAR/EFC protein that induces plasma membrane invagination.. J Biol Chem.

[40] Benesch S, Polo S, Lai FP, Anderson KI, Stradal TE (2005). N-WASP deficiency impairs EGF internalization and actin assembly at clathrin-coated pits.. J Cell Sci.

[41] Qualmann B, Roos J, DiGregorio PJ, Kelly RB (1999). Syndapin I, a synaptic dynamin-binding protein that associates with the neural Wiskott-Aldrich syndrome protein.. Mol Biol Cell.

[42] Braun A, Pinyol R, Dahlhaus R, Koch D, Fonarev P (2005). EHD proteins associate with syndapin I and II and such interactions play a crucial role in endosomal recycling.. Mol Biol Cell.

[43] Qualmann B, Kelly RB (2000). Syndapin isoforms participate in receptor-mediated endocytosis and actin organization.. J Cell Biol.

[44] Soulard A, Lechler T, Spiridonov V, Shevchenko A, Shevchenko A (2002). Saccharomyces cerevisiae Bzz1p is implicated with type I myosins in actin patch polarization and is able to recruit actin-polymerizing machinery in vitro.. Mol Cell Biol.

[45] Otsuki M, Itoh T, Takenawa T (2003). Neural Wiskott-Aldrich syndrome protein is recruited to rafts and associates with Endophilin A in response to epidermal growth factor.. J Biol Chem.

