# Supplementary figures and images for: Regulation of N-WASP and the Arp2/3 Complex by Abp1 Controls Neuronal Morphology
Source: PLoS One. 2007 May 2;2(5):e400. doi: 10.1371/journal.pone.0000400 (PMC1852583; doi:10.1371/journal.pone.0000400)

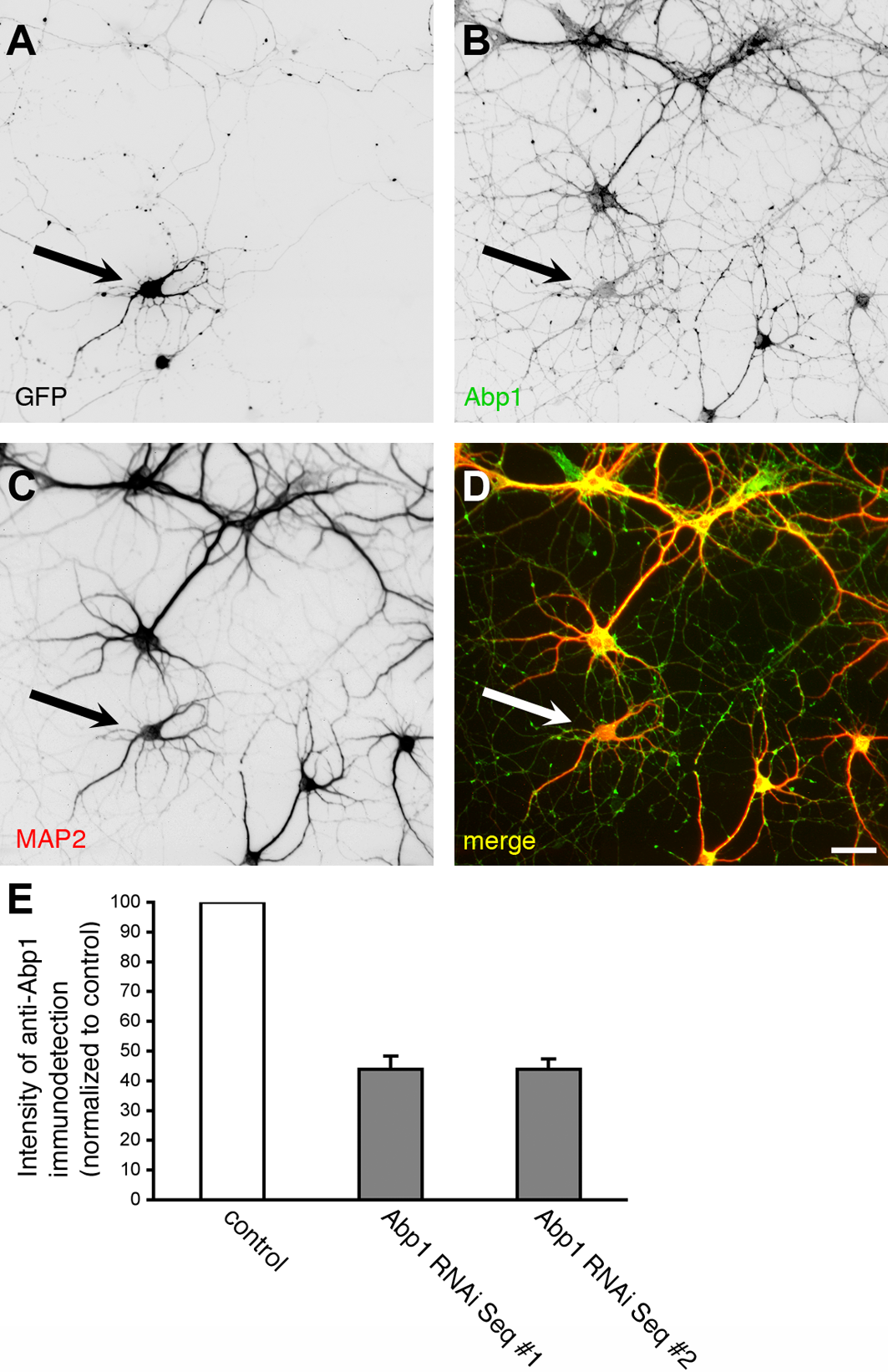

Supplement: Figure S1 — RNAi-based reduction of Abp1 expression levels. (A–D) Primary hippocampal neurons were transfected at day 5 in culture with a vector encoding for GFP and small interfering RNAs complementary to the Abp1 message under two different promotors. Neuronal cells were identified by anti-MAP2 immunostaining (C). Neurons transfected with pRNAT-driven Abp1 RNAi sequence #1 are marked by GFP expression (arrow; A) and showed a significant reduction in the anti-Abp1 immunoreactivity (B). Labelling of images reflects the color of the fluorescence signal in the merged image (D; colocalization appears yellow). (E) Quantitative analysis of the anti-Abp1 immunoreactivity of 50 neurons transfected with pRNAT-driven Abp1 RNAi construct (marked by GFP coexpresion) demonstrates that both Abp1 RNAi sequences tested result in an almost 60% reduction of the anti-Abp1 immuno-fluorescence intensity when compared to the pRNAT control. Data are represented as mean±SEM. Bar = 20 µm. (5.27 MB TIF) [file pone.0000400.s001.tif]

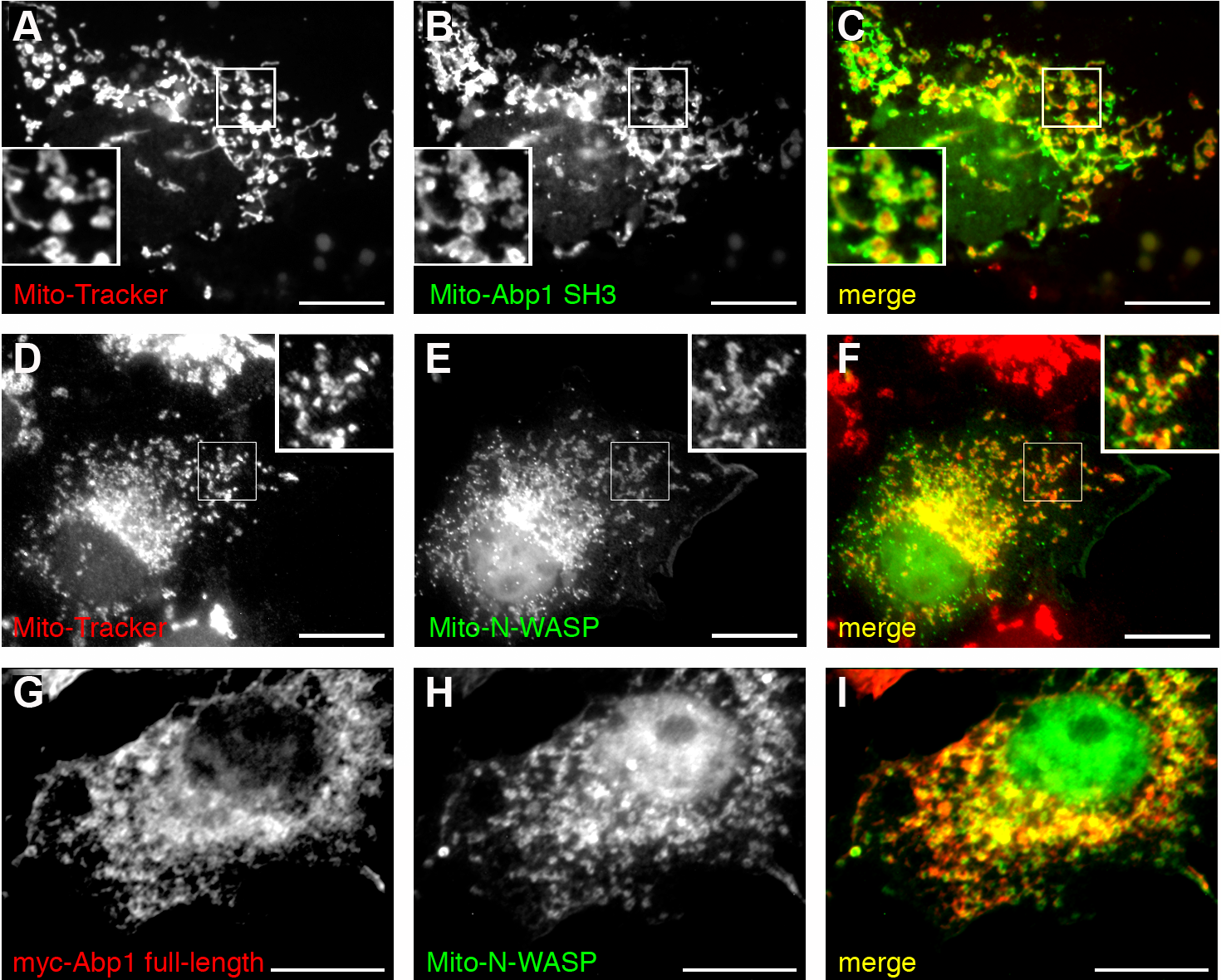

Supplement: Figure S2 — Reconstitution of Abp1/N-WASP complexes at intracellular membranes. N-WASP and Abp1 SH3 domain fusion proteins encompassing a mitochondrial targeting sequence are recruited efficiently to mitochondrial membranes. COS-7 cells were transfected with Mito-GFP-Abp1 SH3 domain (B) and with Mito-GFP-N-WASP (E), respectively. Both Mito-GFP-Abp1 SH3 domain (B) and Mito-GFP-N-WASP (E) were targeted successfully to mitochondria, which were stained with MitoTracker® (A, D). Labelling of images reflects the color of the fluorescence signal in the merged images (C, F, I; colocalization appears yellow). Inserts represent higher magnifications of the boxed areas. Mito-GFP-N-WASP (H) is able to corecruit myc-tagged Abp1 full-length (G) in vivo, as evident by the obtained colocalization on mitochondria (I). Bars (A–F) = 15 µm; bars (G–I) = 10 µm. (5.24 MB TIF) [file pone.0000400.s002.tif]

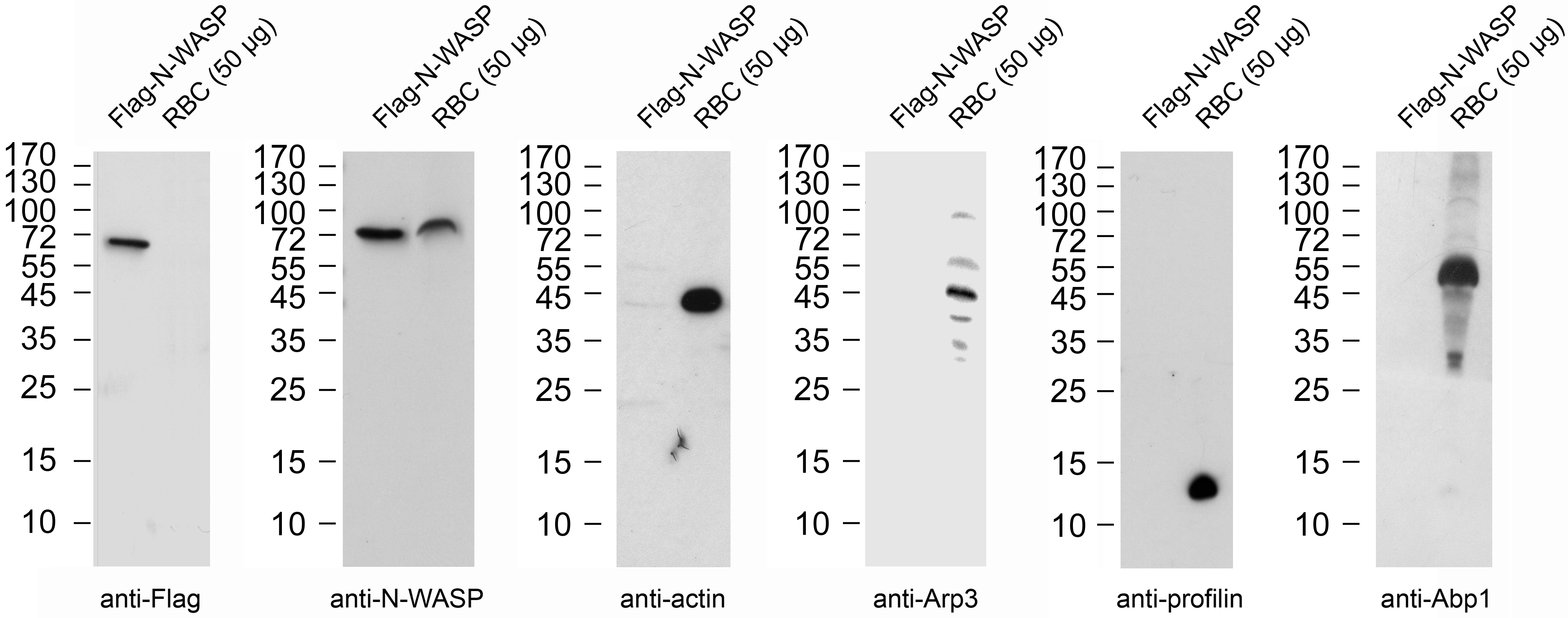

Supplement: Figure S3 — Characterization of immunoisolated Flag-N-WASP by immunoblotting. Flag-tagged N-WASP immunoisolated from COS-7 cells was analyzed further by immunoblotting with different antibodies. Anti-Flag and anti-N-WASP incubations show that the material is intact and of correct size (compare immunosignal of endogenous N-WASP in rat brain extracts (RBC) obtained with anti-N-WASP antibodies). Further analyses demonstrated that the immunoisolation protocol employed is suitable to yield N-WASP material free of direct N-WASP binding partners, such as actin, the Arp2/3 complex component Arp3, profilin (gi|6755040) and Abp1. 50 µg RBC was loaded as positive control for each antibody. (6.39 MB TIF) [file pone.0000400.s003.tif]

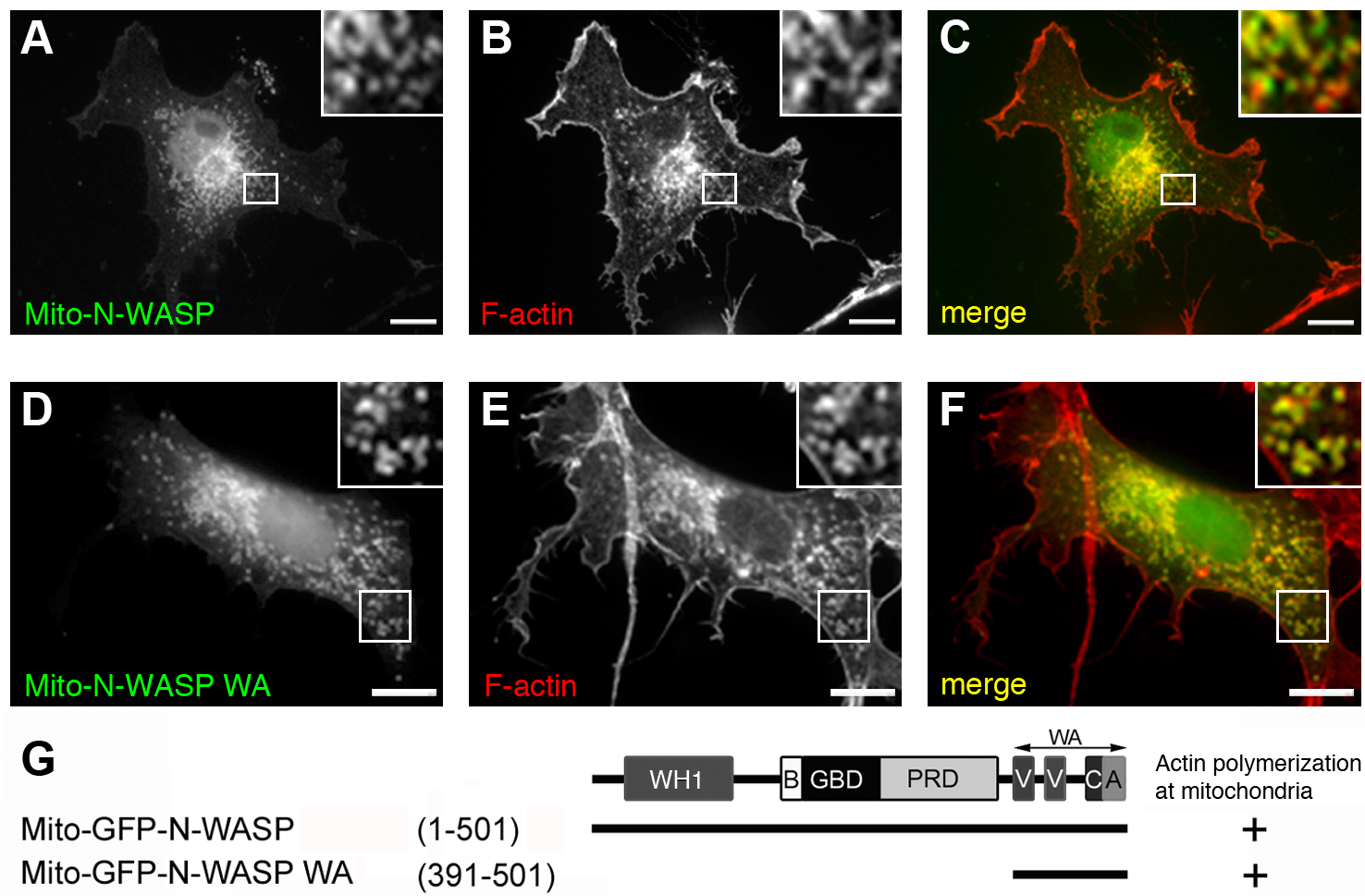

Supplement: Figure S4 — Mito-N-WASP-induced actin polymerization on mitochondrial membranes is mediated by the Arp2/3 complex-interacting C-terminal WA domain COS-7 cells transfected with mitochondrially targeted full-length N-WASP (A) or N-WASP WA (D) showed presence of F-actin polymerized specifically at places of N-WASP targeting, as seen in the Alexa Fluor® 568 phalloidin staining (B, E). Labelling of images reflects the color of the fluorescence signal in the merged images (C and F; colocalization appears yellow). (G) Schematic representation of the parts of the N-WASP protein that trigger Arp2/3 complex mediated actin polymerization at mitochondrial membranes. Bars = 10 µm. (4.32 MB TIF) [file pone.0000400.s004.tif]
